# Small Molecules to Elevate Rab7-GTPase Activity and Lower Cholesterol Accumulation in Niemann-Pick Type C Disease

**DOI:** 10.1007/s11095-026-04058-8

**Published:** 2026-03-03

**Authors:** Mai K. L. Nguyen, Maya R. Nikenich, Kim Seifert, Céline Pinkenburg, Felcia Lai, Hanna-Loisa Walther, Martje Hartmann, Aleksandra Szulc, Eric Sparkes, Shihui Chen, Ravi Wikramanayake, Marc Bernaus-Esqué, Yangjing Liu, Francesc Tebar, Michael Serwetnyk, Anna Wenninger, Christopher Patzke, Brian S. J. Blagg, Brandon L. Ashfeld, Paul W. Groundwater, David E. Hibbs, Andrew J. Hoy, Carles Rentero, Carlos Enrich, Ann-Na Cho, Jonathan J. Du, Thomas Grewal

**Affiliations:** 1 School of Pharmacy, Faculty of Medicine and Health, University of Sydney, Sydney, NSW 2006 Australia; 2 School of Chemistry, Faculty of Science, University of Sydney, Sydney, Australia; 3 School of Biomedical Engineering, Faculty of Engineering, University of Sydney, Sydney, Australia; 4 Departament de Biomedicina, Unitat de Biologia Cel·Lular, Facultat de Medicina I Ciències de La Salut, Universitat de Barcelona, Barcelona, Spain; 5 Fundació de Recerca Clínic Barcelona - Institut d’Investigacions Biomèdiques August Pi I Sunyer (FRCB-IDIBAPS), Barcelona, Spain; 6 Department of Chemistry and Biochemistry, Warren Center for Drug Discovery and Development, University of Notre Dame, Notre Dame, IN USA; 7 Department of Biological Sciences, Boler-Parseghian Center for Rare Diseases, University of Notre Dame, Notre Dame, IN USA; 8 School of Medical Sciences, Faculty of Medicine and Health, University of Sydney, Camperdown, Australia

**Keywords:** late endosome, LDL-cholesterol, NPC disease, Rab7, small molecules, TBC1D15

## Abstract

**Purpose:**

Niemann-Pick type C (NPC) disease caused by mutations in cholesterol transporters NPC1 or NPC2 is characterized by cholesterol accumulation in late endosomes/lysosomes (LE/Lys). The activation of alternative cholesterol export routes that can bypass NPC1/2 deficiency could provide therapeutic opportunities. We previously demonstrated that gene depletion of the Rab7-GTPase activating protein (GAP) TBC1D15, which hydrolyses active GTP-bound Rab7, led to elevated Rab7-GTP levels. This enabled cholesterol export from LE/Lys to reduce cholesterol accumulation in NPC1 mutant cells. Here we aimed to pharmacologically interfere with TBC1D15-mediated Rab7 inactivation to upregulate Rab7 activity and reduce cholesterol accumulation in NPC1 mutant models.

**Methods:**

The protein structure of the GAP domain of human TBC1D15 in complex with human Rab7-GTP served to perform in silico drug screening and identify small molecules with potentially high TBC1D15 binding affinity. Rab-GTP pulldown assays and fluorescence microscopy analyzed the ability of drug candidates to elevate Rab7-GTP levels and reduce cholesterol accumulation.

**Results:**

Four drug candidates reduced cholesterol accumulation in NPC1 mutant Chinese Hamster Ovary (CHO) M12 cells, NPC1 patient fibroblasts as well as differentiated SH-SY5Y neuronal cells and three-dimensional brain organoids treated with U18666A, a pharmacological NPC1 inhibitor. This was associated with elevated Rab7-GTP levels in drug-treated M12 and NPC1 patient fibroblasts. Moreover, drug candidates augmented 2-hydroxypropyl-β-cyclodextrin (HPβCD)-induced cholesterol removal from U18666A-treated SH-SY5Y cells. Notably, drug candidates did not negatively impact on cell viability or cause membrane damage.

**Conclusion:**

Advancing small molecules that can elevate Rab7-GTPase activity could provide opportunities to overcome cholesterol transport defects in NPC mutant cells and offer applications in other Rab7-related neurological diseases.

**Supplementary Information:**

The online version contains supplementary material available at 10.1007/s11095-026-04058-8.

## Introduction

Niemann-Pick type C (NPC) is a neurodegenerative, lethal disorder that occurs due to mutations in the genes encoding cholesterol transporters NPC1 (~ 95%) or NPC2. NPC1/2 deficiency leads to cholesterol accumulation in late endosomes/lysosomes (LE/Lys), compromising membrane trafficking and cellular function, ultimately causing neuronal cell loss and visceral complications [1, 2]. Besides gene deletions and truncations, misfolding and degradation of missense NPC1 mutations (e.g. NPC1^I1061T^) represent the most common gene defects in NPC disease (70–80%).

Treatment options for NPC disease remain limited. Miglustat reduces neuronal glycosphingolipid accumulation and delays the onset of neuronal dysfunction [3]. *N*-Acetyl-L-Leucine (NALL) ameliorates metabolic dysfunction and improves energy metabolism, thereby decreasing neurological symptoms [4]. Heat shock protein (hsp)-based chaperone therapies (Arimocromol) and histone deacetylase inhibitors rescue misfolded NPC1 mutants, which upon drug treatment, reach LE/Lys to correct the NPC cholesterol phenotype [5, 6]. Furthermore, intrathecal delivery of 2-hydroxypropyl-β-cyclodextrin (HPβCD) reduces cholesterol accumulation and slows disease progression in mouse models and several phase 1/2a trials [7, 8]. Recently, intravenous administration of HPβCD also showed clinical benefits in NPC1 patients, with improved cholesterol metabolism in peripheral organs such as the liver, and the ability to cross the blood–brain barrier. Despite noted side effects, these studies revealed potential of HPβCD to reduce cholesterol accumulation in the central nervous system [7–9]. Interestingly, lysobisphosphatidic acid (LBPA) enrichment stimulates late endosomal cholesterol export via pathways that bypass the NPC1 protein [10].

The latter highlights NPC1-independent export routes as an alternative strategy to correct cholesterol transport defects in NPC disease [10–12]. Yet, although other cholesterol transporters exist at the peripheral/limiting LE/Lys membrane, such as Star-related lipid transfer domain-containing 3 (StARD3) and members of the oxysterol-binding protein-related protein family, they appear unable to override NPC1 loss-of-function [2, 11, 13, 14]. In spite of this, previous studies showed ectopic overexpression of several Rab-GTPases, including Rab7, to reduce cholesterol accumulation in LE/Lys of NPC1 mutant fibroblasts [12, 15, 16]. Rab7 is central to late stages of the endocytic pathway, ensuring delivery of Low Density lipoproteins (LDL) to LE/Lys for degradation, and the subsequent distribution of LDL-derived cholesterol from LE/Lys to other organelles. However, cholesterol overload in LE/Lys of NPC1 mutant cells is characterized by low Rab7-GTP levels [12, 17, 18]. Vice versa, genome-wide screens in HeLa and SV589 cells identified that Rab7 gene depletion causes cholesterol accumulation [16, 19, 20]. Of note, Rab7 depletion overcame pharmacological NPC1 inhibition in myeloid K562 cells [21].

We recently showed that in LE/Lys, the scaffold protein annexin A6 (AnxA6) recruits the Rab7-GTPase activating protein (GAP) TBC1D15 to LE/Lys to downregulate Rab7 activity in NPC1 mutant Chinese Hamster Ovary (CHO) lines M12 and 2–2 [12]. Strikingly, gene depletion of TBC1D15 or its scaffold AnxA6 upregulated endogenous Rab7-GTP levels to rescue cholesterol export in NPC1 mutants and enable transfer of cholesterol via the late endosomal cholesterol transporter StARD3 to the endoplasmic reticulum, followed by cholesterol esterification and cholesteryl ester storage in lipid droplets [12].

Gene manipulation approaches remain difficult to progress therapeutically and we therefore aimed to pharmacologically interfere with TBC1D15-mediated Rab7 inactivation, using in silico drug design approaches successfully applied previously [22, 23]. Based on the 3D-structure of the human TBC1D15 GAP domain in complex with human Rab7-GTP, we performed virtual ligand-based screening of a compound library at ‘druggable’ sites at the TBC1D15/Rab7-GTP interface. We identified four drug candidates that reduced cholesterol accumulation in NPC1 mutant M12 and NPC1 patient fibroblasts, which correlated with their ability to elevate Rab7-GTP levels in these cells. Likewise, the lead compounds overcame the blockage of cholesterol export induced by pharmacological inhibition of NPC1 in differentiated SH-SY5Y neuronal cells and 3D-brain organoids. Moreover, drug treatment potentiated HPβCD-induced cholesterol removal from U18666A-treated SH-SY5Y cells. Furthermore, drug-induced restoration of cholesterol export from LE/Lys was associated with reduced expression of autophagy marker proteins, indicating the normalization of dysregulated autophagy in NPC mutants. Drug candidates did not negatively impact on cell viability or cause membrane damage. These drug attributes could overcome NPC1 truncations and deficiency, and also benefit the more prevalent NPC1 missense mutations (e.g. NPC1^I1061T^).

## Material and Methods

### Reagents and Antibodies

Dulbecco's Modified Eagle Medium (DMEM), DMEM/Nutrient Mixture F-12 (DMEM/F-12), Essential 8 and Neurobasal-A medium, Essentiel 8 Supplement, B-27 Supplement, fetal calf serum (FCS), trypsin, Glutamax, penicillin, streptomycin were from Gibco. Bovine serum albumin (BSA), fat-free BSA, 4′,6-diamidino-2-phenylindole (DAPI), dimethylsulfoxide (DMSO), filipin, 2-mercaptoethanol, paraformaldehyde (PFA), retinoic acid (RA), saponin, 12-O-tetradecanoyl-phorbol-13-acetate (TPA), Triton X-100 and Y-27632 were from Sigma. HPβCD was from Genesearch. Mowiol was from Merck. U18666A was from Cayman Chemicals. Gentle Cell Dissociation Reagent, Accutase, Aggrewell 400 6-well plates, and EB Formation Medium were from STEMCELL Technologies. Drug candidates **1–10** were obtained from ASINEX (Gold Platinum library: BAS00402444, BAS00295409, BAS01402908, BAS00434324, BAS01293461, BAS05594113, BAS00512792, ASN08959436, ASN15408181, BAS03450598).

Rabbit polyclonal anti-AnxA6 was prepared in our laboratory and has been described previously [24, 25]. Rabbit polyclonal anti-NPC1, anti-TBC1D15, anti-LDL receptor and mouse monoclonal anti-GST were from Abcam. Rabbit monoclonal anti-Rab7 and polyclonal anti-β-actin were from Cell Signaling. Alexa-488, Alexa-594 and Cy5-conjugated secondary antibodies were from Life Technologies. Horseradish peroxidase (HRP)-labelled secondary antibodies and SDS-PAGE molecular weight markers were from Cell Signaling. Lipoprotein-deficient serum (LPDS) from FCS was prepared by preparative ultracentrifugation [12], dialyzed extensively against PBS and stored at 4 °C until use. Expression vectors encoding mRFP-Rab7a and GFP-Rab7 were from Ari Helenius (ETH Zürich, Switzerland; Addgene #14,437) [26] and Michiyuki Matsuda (University of Kyoto, Japan), respectively. The expression vector encoding EYFP-TBC1D15 was generated in our laboratories [12]. Glutathione S-transferase (GST) fusion proteins (Rab interacting lysosomal protein (RILP)-C33-GST, GST-perfringolysin (GST-PFO)) were produced in *E.coli BL21* cells and purified using glutathione Sepharose 4B beads (GE Healthcare) as described [12].

### Protein Structure Modelling and in Silico Drug Screening

The protein sequences of the GAP domain of the human TBC1D15 (301–631) and full length human Rab7a (1–185) were taken from Uniprot [27] and served as input into LocalColabFold [28, 29] to produce the human TBC1D15/Rab7 complex. The TBC1D15 protein was extracted from the protein complex and used as input for the molecular modelling studies. All preparation and modelling studies were performed utilizing Schrödinger software [30]. Protein preparation was carried out using the Protein Preparation Wizard [31], which included adding hydrogens, assigning bond orders and generating disulfide bonds. The hydrogen bond network was then optimised and the protein structure minimised to a root mean square deviation of 0.3 Å with the OPLS4 forcefield [32]. The SiteMap tool [33, 34] was employed to identify potential drug binding sites on the TBC1D15 protein, which were chosen based on their proximity to the area which also interacts with the Rab7 protein. The Receptor Grid Generation tool in Glide [35, 36] then served to characterize the site for docking studies, using the binding site identified by the SiteMap tool bound by a 20 × 20x20Å^3^ bounding box. The ligands from the ASINEX Gold Platinum library (261,120 compounds) were prepared with the Ligand Preparation Wizard and involved geometry minimisations based on the OPLS4 forcefield [32]. The molecular docking was carried out in a 3-stage process utilizing the Glide docking software. The ASINEX library was first screened with the High-Throughput Virtual Screening mode. The top 30% of hits were taken and screened with Single Precision mode. The top 20% of hits were then screened with the Extra Precision mode. The top 10 hits sorted by Glide gScore were then taken for experimental testing.

### Cell Culture

CHO M12 cells were kindly provided by Laura Liscum (Tufts University School of Medicine, USA). CHO-WT and SH-SY5Y cell lines were obtained from the European Collection of Authenticated Cell Cultures (ECACC: #85,051,005) and American Type Culture collection (ATCC: CRL2266), respectively. The generation of the CRISPR/Cas9-edited CHO M12 cell line lacking AnxA6 (CHO M12-A6ko) has been described [12]. NPC1 patient fibroblasts (GM22871, GM03123, GM18453) and control fibroblasts (GM05659) were obtained from Coriell Cell Repositories (Coriell Institute).

CHO-WT, CHO M12, CHO M12-A6ko and SH-SY5Y cells were grown in DMEM/F12, NPC1 patient and control fibroblasts were grown in DMEM, together with 10% FCS, L-glutamine (2 mM), penicillin (100 U/ml) and streptomycin (100 µg/ml) at 37 °C, 5% CO_2_. For the differentiation of SH-SY5Y cells into a neuronal phenotype, cells were grown for 3 days with 10 μM RA in media containing 2.5% FCS, followed by 3 days in media containing 10 μM RA and 81 nM TPA as described [37]. Differentiation was monitored by microscopy and confirmed by phenotypic axonal projections. For transient transfections with fluorescently-tagged (EGFP, mRFP, EYFP) Rab7 and TBC1D15, cells at 50% confluence were incubated with 1.5 μg DNA/ml using Lipofectamine 2000 following manufacturer’s instructions [38].

### Suppression of Rab7a Expression

1–2 × 10^6^ CHO M12 and SH-SY5Y cells were transfected with 100 nM ON-TARGET plus SMARTpool siRNA (Dharmacon) targeting human Rab7a mRNA (NM_004637) at position 480–498 (5’-CUAGAUAGCUGGAGAGAUG-3’), 715–733 (5’-AAACGGAGGUGGAGCUGUA-3’), 385–353 (5’-GAUGGUGGAUGACAGGCUA-3’) and 243–261 (5’-GGGAAGACAUCACUCAUGA-3’), together with Lipofectamine 2000 as described [39] After transfection, cells were grown in media containing 10% LPDS for 48 h, then loaded with 10% FCS ± 10 μM (CHO M12) or 50 μM (SH-SY5Y) drug candidate **10**. Rab7 depletion was confirmed by western blotting. CHO M12 and SH-SY5Y cells expressing scrambled siRNA (5’-GGAAUCUCAUUCGAUGCAUAC-3’) served as negative controls.

### Fluorescence Microscopy

Cells grown on coverslips were fixed with 4% PFA for 30 min at room temperature (RT), washed with PBS, permeabilized with 0.05% saponin, 0.5% glycine in PBS for 5 min and blocked with 2% BSA in PBS (0.05% saponin, 0.5% glycine) for 30 min. Free cholesterol was stained with 0.05 mg/ml filipin for 60 min, washed and mounted in Mowiol as described [12].

Alternatively, cellular cholesterol was stained with recombinant GST-PFO. Cells were fixed in 4% PFA for 30 min and washed with PBS. Cells were permeabilized with 0.1% Triton X-100 for 5 min, washed extensively with PBS, and blocked with 3% fat-free BSA in PBS for 30 min at RT. Cells were incubated with 10 μg/ml purified recombinant GST-PFO in blocking buffer for 1 h at RT, washed with blocking buffer, followed by incubation with primary antibody (mouse anti-GST, 1/200) diluted in blocking buffer for 1 h at RT. Coverslips were washed intensively with blocking buffer and then incubated with the adequate Alexa-488, Alexa-594 or Cy5-conjugated secondary antibodies for 45 min at RT. After staining, coverslips were washed with PBS or DAPI-PBS solution and mounted in Mowiol. Samples were visualized using a Leica DMI6000B epifluorescence inverted microscope equipped with an HCX PLA APO 63 × oil immersion objective lens or a Zeiss upright AXIO Scope.A1 fluorescence microscope equipped with a 40 × objective lens. Image analysis was performed with ImageJ2 (version: 2.16.0). For the quantification of filipin and GST-PFO staining intensity after different treatments, images were captured and systematically screened and analyzed using identical microscope settings. Regions of interest (e.g. perinuclear region for filipin and anti-GST puncta) were selected, images were quantified, excluding the nuclear area, and corrected for background fluorescence.

### Western Blot Analysis

Cells were lysed in lysis buffer (50 mM Tris–HCl, 150 mM NaCl, 1% Triton X-100, 0.1 mM CaCl_2_, pH 7.4) supplemented with protease/phosphatase inhibitors cocktail (1 mM Na_3_VO_4_, 10 mM NaF, 1 mM phenylmethylsulfonyl fluoride (PMSF), 10 µg/ml leupeptin, 10 µg/ml aprotinin). Lysates were boiled in 1 × sample buffer, resolved on 10–12% SDS-PAGE and transferred to Immobilon-P (Millipore) membranes as described [38]. Membranes were blocked in 5% non-fat milk, incubated overnight in primary antibodies, washed in TBS-T, incubated with HRP-conjugated secondary antibodies and developed using enhanced chemiluminescence detection (ECL, Perkin-Elmer) [38]. ImageJ2 software was used for quantitative analysis of WB bands.

### GST Pulldowns

5 × 10^6^ cells were grown in media containing 10% LPDS for 48 h, followed by incubation with 10% FCS ± 50–200 μM drug candidates (**3**, **6**, **9**, **10**) or 0.1% DMSO for 2 h at 37 °C, 5% CO_2_. Organoids grown in 96-wells were incubated with 10% FCS ± U18666A (2 mg/ml) for 48 h. Cells or organoids were solubilized in pull-down buffer (50 mM Tris pH 7.3, 150 mM NaCl, 1% Triton X-100, 0.1 mM CaCl_2_ plus protease/phosphatase inhibitors as above) and centrifuged at 14,000 rpm for 10 min at 4°C. The pellet was discarded and 700 μg protein of the supernatant was incubated with glutathione Sepharose 4B beads coated with recombinant 100 μg RILP-C33-GST protein for 2 h at 4 °C as described [12]. Samples were washed three times and collected in 40 μl 1 × loading buffer. Rab7-GTP bound to the beads and total Rab7 from whole cell lysates was analyzed by western blotting and the ratio of Rab7-GTP/Total Rab7 was calculated. In some experiments, the amount of TBC1D15 in the Rab7-GTP fraction was also analyzed.

### Subcellular Fractionation

1 × 10^7^ cells were grown in media containing 10% LPDS for 48 h, followed by incubation with 10% FCS ± 200 μM drug candidate **10** or 0.1% DMSO for 2 h at 37 °C, 5% CO_2_. Cells were washed with cold PBS and scraped in 500 μl buffer A (10 mM Tris–HCl, pH 7.5; 5 mM MgCl_2_, 1 mM EGTA, 1 mM DTT plus protease inhibitors as above). Cells were homogenized with 20 passages through a 23-gauge needle and nuclei were removed by low-speed centrifugation. The postnuclear supernatants were spun at 100,000 g at 4 °C for 30 min as described [40]. The supernatants containing cytosolic proteins were collected and the pellets containing cellular membranes were resuspended in 100 μl buffer A. The protein content was determined and 30 μg of membrane and cytosolic fractions were analysed by western blotting for TBC1D15, LDL receptor and β-actin.

### Cell Cytotoxicity

5 × 10^3^ cells were seeded in 96-well plates and grown in LPDS-containing media for 48 h, followed by 24 h in media supplemented with FCS ± drug candidates (500 nM –250 μM). 0.1% DMSO served as negative control. Cell viability was determined using the colorimetric CellTiter 96® AQueous Cell Proliferation Assay Kit (MTS, 3-(4,5-dimethylthiazol-2-yl)−5-(3-carboxymethoxyphenyl)−2-(4-sulfophenyl)−2H-tetrazolium; PMS, phenazine methosulfate) according to the manufacturer’s (Promega) instructions [41]. Lactate dehydrogenase (LDH) assays served to determine cell membrane damage, using the CyQUANT™ LDH Cytotoxicity Assay (Invitrogen) as described [42]. The absorbance of samples at 550 nm (MTS) and 490 nm (LDH) was measured in a Bio-Rad Model 680 microplate reader. Reactive oxygen species (ROS) levels were determined using the general oxidative stress indicator CM-H2DCF-DA (diluted 1/2000 in PBS) as per manufacturer’s instructions (Invitrogen) [42]. The fluorescence of cells was measured using a PerkinElmer VictorTM X4 Multimode plate reader at 485/535 nm excitation/emission wavelengths. All experiments were performed in triplicate in three independent experiments.

### Human Embryonic Stem Cell Maintenance and Brain Organoid Generation

The generation of human dorsal organoids followed the protocol described in [43] with minor modifications. Human embryonic stem cells (H9, WiCell; between 40–55 passages) were maintained on Matrigel-coated 6-well plates (hESC-qualified) in Essential 8 medium supplemented with Essentiel 8 Supplement. At ~ 80% confluence, cells were dissociated to single-cell suspensions using Accutase. Approximately 8–9 × 10^3^ cells were seeded into AggreWell 400 6-well plates in EB Formation Medium supplemented with 50 µM ROCK inhibitor Y-27632 to facilitate embryoid body (EB) formation. After 24 h, the formed EBs were carefully transferred to ultra-low attachment dishes to prevent clumping and enable uniform growth. Neuroepithelial differentiation was induced by culturing organoids in Neurobasal-A medium supplemented with penicillin (100 U/ml), streptomycin (100 µg/ml), 1% Glutamax and B-27 Supplement without vitamin A (1:50, v/v). Organoids were matured for at least 45 days before incubation for 48 h ± 10% FCS and U18666A (2 μg/ml) at 37 °C, 5% CO_2_ (see also GST pulldowns), respectively, followed by the addition of 50 μM compound (**3**,** 6**, **9**, **10**) for additional 24 h.

To stain organoids for cellular cholesterol, they were collected in low-binding (LB) tubes, washed with PBS and fixed with 4% PFA for 30 min at RT. Following this, organoids were washed three times with PBS and permeabilized with 0.5% Triton X-100 for 30 min at RT. Next, organoids were blocked with 3% fat-free BSA for 5 h at RT and then incubated with 10 μg/ml GST-PFO in PBS overnight. Organoids were washed with PBS, incubated with mouse anti-GST (1:500 diluted in PBS) at 4 °C for 2 days, washed with PBS for 6 h, followed by addition of Alexa-488 conjugated secondary antibody (1:100 diluted in PBS) at 4 °C for 2.5 days. Organoids were washed 3 times with PBS, DAPI/PBS solution was applied at RT for 30 min and after another wash with PBS, analyzed by confocal microscopy. High-resolution imaging was performed using an Olympus FV3000 laser scanning confocal microscope equipped with a 40 × objective. All images were acquired under consistent laser and constant exposure times across the various experimental groups [44].

### Cholesterol Determination

5 × 10^5^ SH-SY5Y cells in 6-well plates were grown in media supplemented with 10% LPDS for 48 h before addition of 10% FCS ± U18666A (2 μg/ml) for additional 24 h. The media was removed, cells were washed and lipids were extracted as described [45] and cholesterol was measured using the Amplex™ Red Cholesterol Assay Kit (Molecular Probes) [46, 47].

Alternatively, SH-SY5Y, CHO-M12 and CHO-WT cells were grown in media supplemented with 10% LPDS for 48 h, followed by 48 h in 10% FCS ± U18666A (2 μg/ml) and treatment ± 50 μM drug candidate **10**, respectively. Then the media was removed, cells were washed with PBS and incubated in serum-free media ± 1% HPβCD for 60 min. Cells were washed, lipids were extracted and cholesterol was measured as described above.

### Statistics

Statistical analysis was carried out using Microsoft Excel and GraphPad Prism 10.6. Unless stated otherwise, data represent the mean of at least two independent experiments with duplicate or triplicate samples in each experiment, and error bars show the standard deviation (SD). Statistical comparison was performed using one-way ANOVA followed by Dunnett’s post-hoc test or unpaired Student’s *t* test as required. * = *p* < 0.05, ** = *p* < 0.01, *** = *p* < 0.001, **** = *p* < 0.0001.

## Results

### Virtual Modelling Identifies Candidate Molecules that Can Bind at the Interface of the TBC1D15/Rab7 Complex

**Fig. 1:**
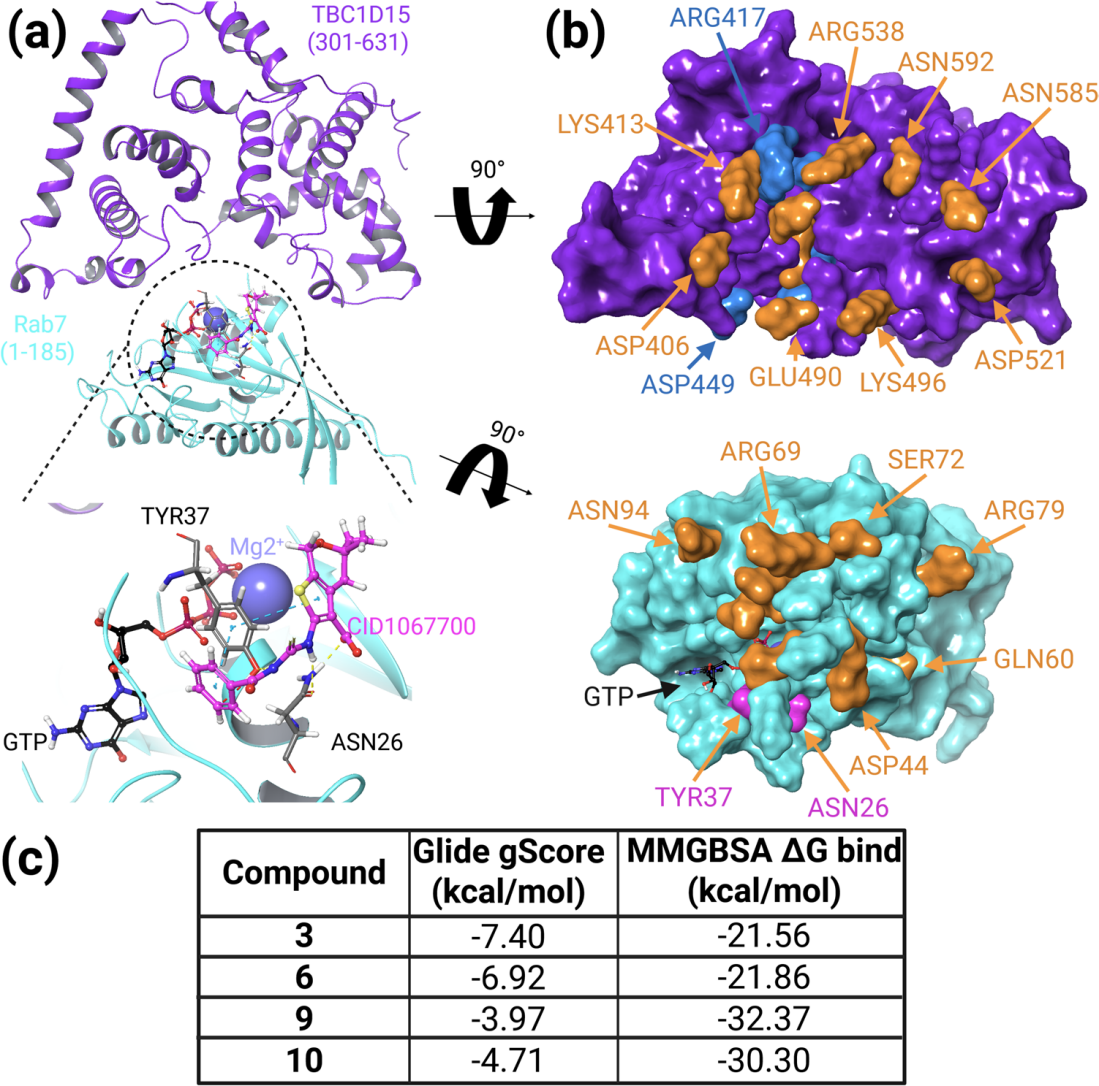
In silico modelling of the human TBC1D15 GAP domain/Rab7-GTP complex and identification of drug candidates. **(a)** TBC1D15 (301–631) (purple) in complex with Rab7 (1–185) (cyan) with known Rab 7 inhibitor CID1067700 (pink carbons) bound to the exterior surface of Rab7 in conjunction with GTP (black carbon) and magnesium ion. Hydrogen bonds are denoted with yellow dashed lines and π-π interactions are denoted by cyan dashed lines. **(b)** Structure of the TBC1D15 and Rab7 proteins with surface representation shown. Orange residues denote amino acids involved in the interaction between TBC1D15 and Rab7, while dark blue residues indicate areas where the lead compounds bind. Pink residues represent residues which interact with CID1067700 on Rab7. **(c)** Summary of the molecular modelling results (Glide gScore, Molecular Mechanics with Generalized Born and Surface Area (MMGBSA) of the lead compounds **3** (CAS 1397056–96-0), **6** (CAS 400749–204), **9** (CAS 1011363–79-3) and **10** (CAS 431980–31-3) discussed in the manuscript (for chemical structure see Suppl. Fig. 1).

Using gene depletion approaches, we recently demonstrated that the loss of TBC1D15-mediated Rab7 inactivation elevated Rab7-GTP levels, enabling cells to bypass NPC1 deficiency and establish NPC1-independent transport routes to overcome inhibition of cholesterol egress from LE/Lys [12]. To identify drug candidates that could pharmacologically interfere with TBC1D15-mediated Rab7 inactivation, we used in silico drug design [22, 23] and first modelled the 3D-structure of the human TBC1D15 GAP domain (purple) in complex with human Rab7-GTP (cyan) (Fig. 1a). The computationally created TBC1D15/Rab7 complex aligned with the 3D-model obtained from the crystal structures of the Shark and Sus TBC1D15 GAP domains bound to human Rab7a [48]. In support of this complex reflecting a suitable model for virtual drug screening and computational drug design, the ability of the Pan Rab-GTPase inhibitor CID-1067700 (pink) to competitively inhibit GTP binding of Rab7 is indicated by their close proximity when superimposed on the TBC1D15/Rab7 complex (Fig. 1a) [49].

Next, several potentially ‘druggable’ sites at the TBC1D15/Rab7-GTP interface were identified (Fig. 1b), followed by virtual ligand-based in silico screening of a compound library (> 260,000 compounds) enriched with small molecules characterized by their high degree of drug-likeness in accordance with Lipinski’s rule of five [50]. This guideline predicts that orally active drugs ideally violate not more than one of the following four rules: a molecular mass less than 500 Da, a logP (lipophilicity) under 5, and no more than 5 and 10 hydrogen bond donors and acceptors, respectively. Figure 1b shows the surface representation of the TBC1D15 and Rab7 complex when rotated 90 degrees with residues involved in the interaction between TBC1D15 and Rab7 (orange), while neighboring residues that interact with potential lead compounds are shown in blue. Interestingly, in the lower panel, Rab7 residues that interact with CID1067700 (pink) are in close proximity to several TBC1D15 and Rab7 interacting residues. Based on scoring functions that approximate the ligand binding free energy (Glide gScore; Molecular Mechanics with Generalized Born and Surface Area, MMGBSA) [51], we listed four of the top 10 drug candidates identified in this screen with potentially high TBC1D15 binding affinities (Fig. 1c; for chemical structure see Supplementary Fig. S1).

### Screening of Small Molecules to Reduce Cholesterol Accumulation in NPC1 Mutant CHO M12 Cells

We then first assessed the ability of all top 10 drug candidates to reduce cholesterol accumulation at steady-state conditions when cholesterol-loaded NPC1 mutant M12 cells continuously grow in lipid-rich media. These cells lack the NPC1 protein and compared to wildtype (WT) controls, express comparable levels of TBC1D15 and Rab7 (Supplementary Fig. S2a). Cells were grown in media containing 10% FCS and incubated for 24 h with either DMSO (0.1%) or 5–50 μM compound **1–10**. Cells were fixed and free cholesterol was stained with filipin as described [12]. Representative images of M12 cells incubated ± DMSO or compounds **3** and **8** are shown (Supplementary Fig. S2b). In these initial screening assays, compounds **3**, **6**, **9** and **10** showed an ability to reduce filipin staining intensity by ~ 20–35% (see quantification in Supplementary Fig. S2c). Based on the root mean square deviation [51] and the Tanimoto similarity coefficient [52], these four compounds lacked similarities to approved medications for NPC1 disease (Miglustat, NALL, Arimoclomol), other cholesterol-reducing drug candidates (HPβCD, LBPA), the 21 compounds identified in previous screens to overcome the cholesterol transport defect in NPC1 mutants [53], or thioperamide and other histamine receptor H3 inhibitors, which elevate LBPA levels to lower late endosomal cholesterol in NPC1 patient fibroblasts [54].

**Fig. 2:**
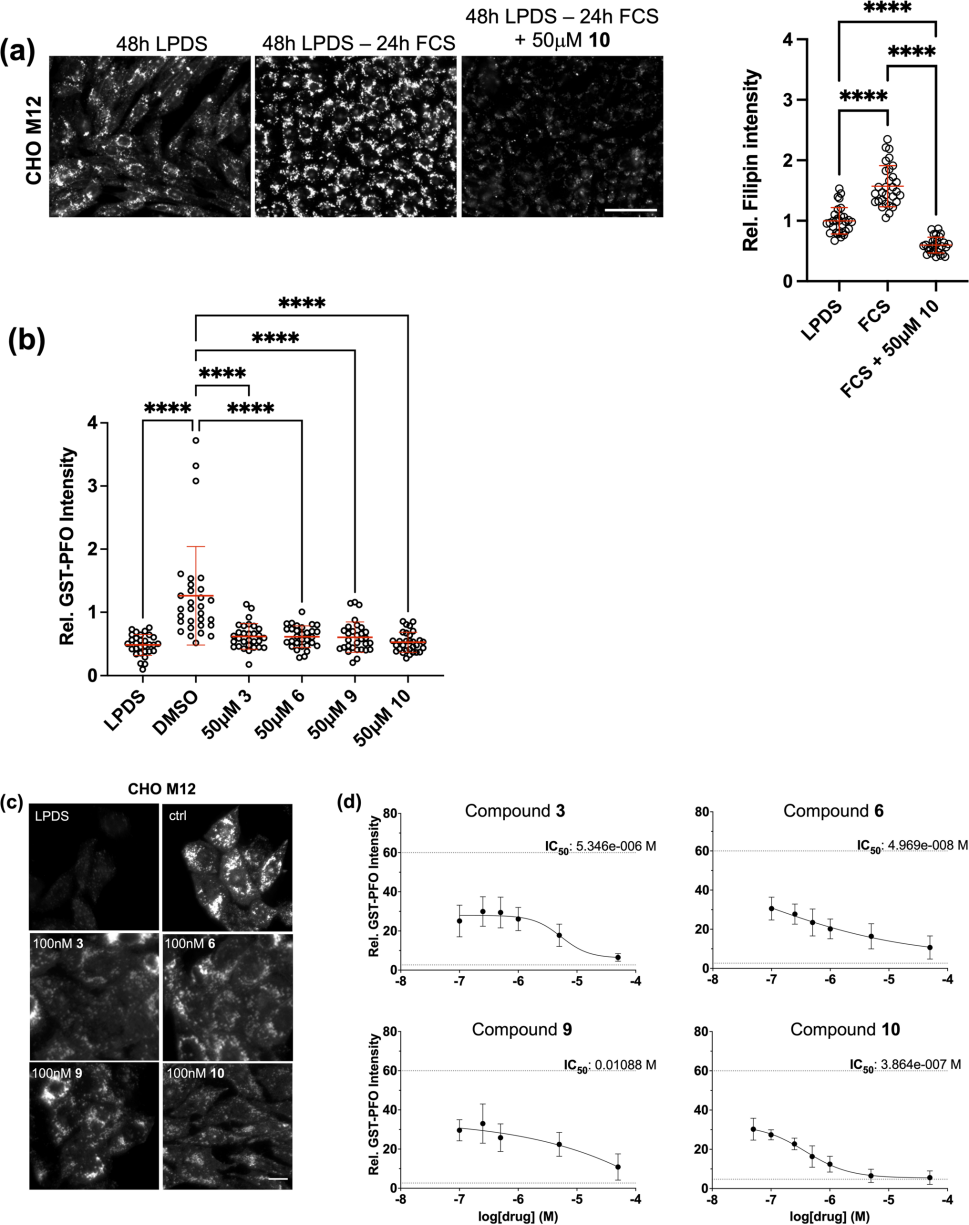
Identification of small molecules that reduce cholesterol accumulation in NPC1 mutant CHO M12 cells. **(a-b)** CHO-M12 cells on coverslips were grown in 10% lipoprotein-deficient serum (LPDS) for 48 h. After lipid depletion, cells were grown in 10% fetal calf serum (FCS) ± 50 μM compound **3**, **6**, **9** and **10** for 24 h. Cells were fixed and free cholesterol was stained with **(a)** filipin or **(b)** GST-PFO (see Methods for details). Representative images are shown. Bar is 100 μm. **(c-d)** CHO-M12 cells were grown ± 50 nM—50 μM compound **3**, **6**, **9** and **10** for 24 h, fixed and stained with GST-PFO as indicated. Representative images of starved cells (LPDS) and cells incubated with DMSO (ctrl) or 100 nM compound **3**, **6**, **9** and **10** are shown. **(a-d)** In each experiment, 5–8 images from each condition were captured at identical settings with fixed intensities below their saturation. Fluorescence intensity (30 cells/condition from 5–6 images) was determined using NIH ImageJ2 software. GraphPad Prism 10.6 was used for statistical analysis. In panel D, the IC_50_ values from three independent experiments are given and the staining intensity of starved and DMSO-treated cells is indicated (dotted lines). The mean and standard deviation (SD) is shown. One-way ANOVA followed by Dunnett’s post-hoc test was used to determine statistical significance. **** *p* < 0.0001. Bar is 10 μm.

To better monitor the fate of internalized cholesterol once it reaches LE/Lys in drug-treated NPC1 mutants and further reduce endogenous cholesterol contributing to background staining, cells were lipid-starved in 10% LPDS for 48 h, prior to cholesterol loading with 10% FCS in the presence or absence of 50 μM lead compound **10** for additional 24 h. As expected, the 48 h lipid starvation was characterized by low cholesterol staining intensity in M12 cells, which increased > 1.5-fold with subsequent loading with 10% FCS due to late endosomal/lysosomal cholesterol accumulation. Impressively, supplementation with compound **10** strongly reduced filipin staining intensity in FCS-incubated M12 cells (~ 2.6-fold compared to control) (Fig. 2a).

Based on these findings, we next aimed to characterize the concentration-dependent cholesterol-lowering properties of the lead compounds **3**, **6**, **9** and **10**. Yet, the quantitative detection of cellular cholesterol using filipin can be challenging as rapid photobleaching of filipin fluorescence compromises reproducibility. Alternatively, the domain 4 of the cholesterol-binding toxin PFO fused to GST allows binding to cholesterol in fixed and permeabilized cells, which enables quantification of cellular cholesterol by fluorescent immunodetection of GST [12, 55]. This method is highly sensitive and does not suffer from photobleaching, ensuring reproducibility when comparing large sample collections over multiple experiments [56]. Thus, cells were starved in 10% LPDS for 48 h, followed by 24 h cholesterol loading with 10% FCS ± 50 μM of compounds **3**, **6**, **9** and **10**. Then cells were fixed, incubated with GST-PFO and stained with anti-GST. Using this approach, 50 μM of compounds **3**, **6**, **9** and **10** reduced GST-PFO staining intensity 2—2.5-fold compared to DMSO-incubated M12 cells (Fig. 2b) and similar to culturing M12 cells for 48 h in the absence of lipoproteins (LPDS). We next performed dose titrations (50 nM – 50 μM) (Fig. 2c-d) and at 100 nM, all four lead compounds significantly reduced cholesterol staining intensity by 30–48%, respectively. The IC_50_ values for lowering cholesterol accumulation for three of the four compounds (**3**, **6**, **10**) were in the mid-nanomolar to low-micromolar range. At these concentrations (< 5 μM), cell viability as determined by MTS and LDH assays, was not significantly affected (Supplementary Fig. S3a). Likewise, ROS assays in cells incubated with 500 nM – 250 μM lead compounds determined oxidative stress levels comparable to 0.1% DMSO-treated controls (Supplementary Fig. S3a). Also, expression levels of TBC1D15 and Rab7 upon drug exposure were comparable to untreated controls (Supplementary Fig. S3b) and cellular uptake of cholesterol was not compromised by compounds **6** and **9** (Supplementary Fig. S3c). Taken together, drug candidates targeting the TBC1D15/Rab7 interface stimulate cholesterol removal from NPC1 mutant cells without compromising cell viability or cellular cholesterol uptake.

### Small Molecules Targeting the TBC1D15/Rab7 Interface Elevate Rab7-GTP Levels

**Fig. 3:**
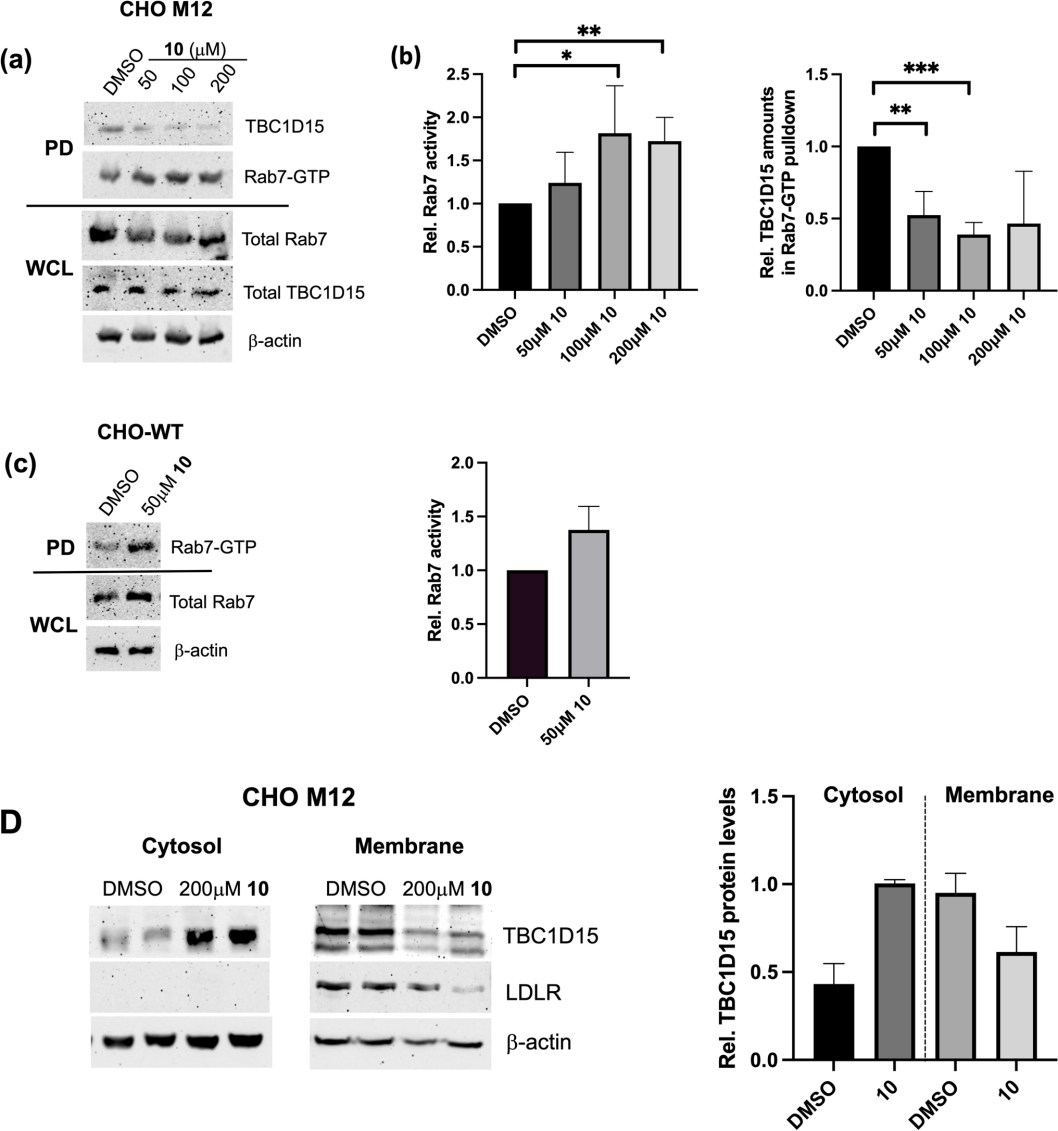
Small molecules targeting the TBC1D15/Rab7 complex increase Rab7-GTP levels in NPC1 mutant CHO M12 cells. **(a-b)** CHO M12 and **(c)** CHO wildtype (WT) cells were grown in 10% LPDS for 48 h, followed by lipid-loading in 10% FCS ± 50–200 μM compound **10** for 2 h as indicated. Whole cell lysates (WCL) were prepared and Rab7-GTP pulldown (PD) assays were performed as described (see Methods for details). The amounts of active (Rab7-GTP)**,** TBC1D15 associated with Rab7-GTP in pulldowns and the protein levels of total Rab7, TBC1D15 and β-actin in WCL were determined by western blotting and quantified. Representative western blots from pulldowns and WCL of **(a)** M12 cells and **(c)** CHO-WT are shown. ImageJ2 software was used for quantitative analysis of WB bands from four independent experiments. GraphPad Prism 10.6 was used for statistical analysis (unpaired Student’s t-test). * p,0.05, ** *p* < 0.01, *** *p* < 0.001. **(d)** CHO M12 cells were grown in 10% LPDS for 48 h, followed by lipid-loading in 10% FCS ± 200 μM compound **10** for 2 h as indicated. Whole cell lysates were prepared and subjected to subcellular fractionation (see Methods for details). Membrane and cytosolic fractions were analyzed by western blotting for the amounts of TBC1D15, LDL receptor and β-actin. The presence of LDL receptor in membrane, but not cytosolic fractions, confirmed the purity of subcellular fractions. A representative western blot from two independent experiments is shown. ImageJ2 software was used for quantitative analysis of WB bands from two independent experiments. The mean and standard deviation (SD) is shown.

Given the potential of the four drug candidates to reduce cholesterol accumulation in NPC1 mutants via interfering with TBC1D15/Rab7 assembly, we next determined if this was associated with increased Rab7-GTP levels in drug-treated M12 cells. Therefore, we performed RILP-C33-GST pulldown assays that allow determination of Rab7-GTP levels based on the specific binding of Rab7-GTP to a truncated form of the Rab7 effector RILP (Rab7 interacting lysosomal protein) [12]. M12 cells were grown in 10% LPDS for 48 h, followed by lipid-loading in 10% FCS in the presence or absence of 50–200 μM compound **10** for 2 h. Lysates were prepared, subjected to RILP-C33-GST pulldown assays and analyzed by western blotting to determine Rab7-GTP amounts (Fig. 3a-b). Strikingly, compound **10** significantly elevated Rab7-GTP levels (~ 30–80%), which was associated with a substantial reduction in TBC1D15 amounts in complex with active Rab7-GTP (30–60%). Likewise, compound **10** elevated Rab7-GTP levels in WT cells (Fig. 3c). In line with these findings, annexin A6 (AnxA6)-deficient M12-A6ko cells, which show reduced TBC1D15 membrane association and loss of TBC1D15-mediated Rab7 inactivation due to the lack of the AnxA6 scaffold protein [12], displayed a ~ 1.5-fold elevation of Rab7-GTP levels (Supplementary Fig. S4a).

To assess if the Rab7-stimulating capacity of compound **10** correlated with reduced TBC1D15 membrane association, we performed subcellular fractionations and prepared membrane and cytosol fractions of M12 cells incubated with and without 200 μM compound **10** for 2 h. Fractions were separated by 12% SDS-PAGE and western blotting for the LDL receptor confirmed the purity of membrane and cytosol fractions (Fig. 3d). In DMSO-treated control cells, TBC1D15 was predominantly associated with membranes (~ 70%). Yet, upon incubation with compound **10**, the majority of TBC1D15 proteins were in the cytosolic fractions (> 60%), indicating that compound **10** interfered with TBC1D15 membrane recruitment, with potential consequences for TBC1D15/Rab7 assembly in LE/Lys and other organelles.

### Drug Candidates Reduce Cholesterol Accumulation and Elevate Rab7-GTP Levels in NPC1 Patient Fibroblasts

**Fig. 4:**
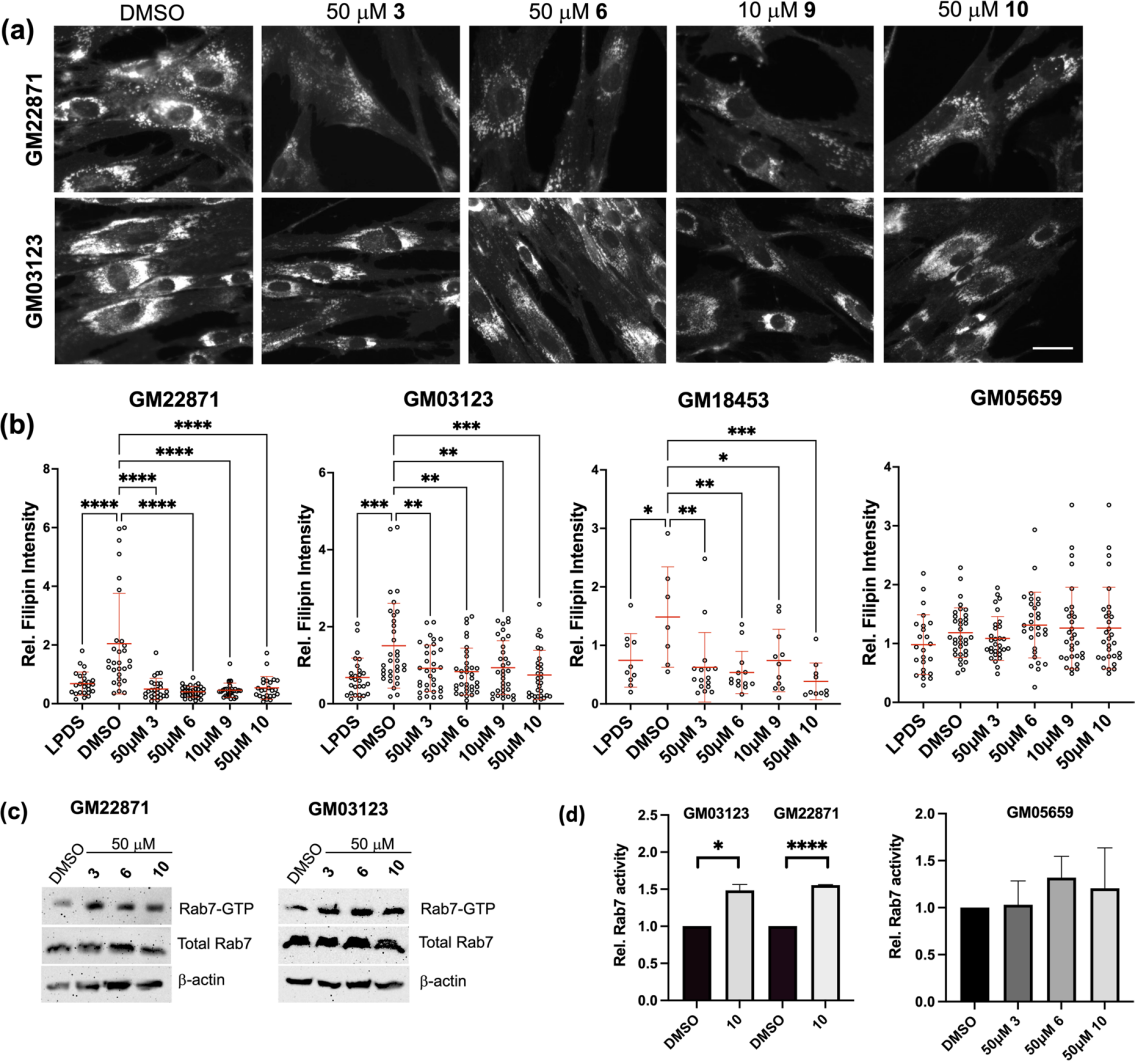

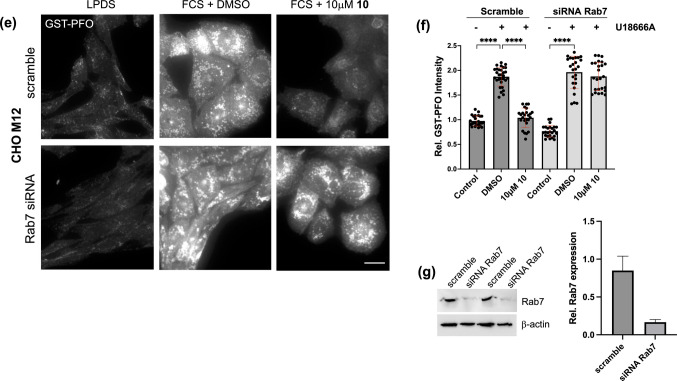
Drug candidates reduce cholesterol accumulation and elevate Rab7-GTP levels in NPC1 patient fibroblasts. **(a-b)** NPC1 patient (GM22871, GM03123, GM18463) and control fibroblasts (GM05659) were starved for 48 h, incubated in 10% FCS ± 10 μM compound **9**, 50 μM compound **3**, **6** and **10** or DMSO alone for 24 h. Cells were fixed and free cholesterol was stained with filipin (see Methods for details). Representative images of NPC1 patient fibroblasts GM22871 and GM03123 incubated with DMSO or compounds **3**, **6**, **9** and **10** are shown and are representative for three independent experiments. In each experiment, 5–8 images from each condition were captured at identical settings with fixed intensities below their saturation and the staining intensity of starved (LPDS), DMSO- and drug-treated cells was calculated. Fluorescence intensity (24–34 cells/condition from 5–7 images from GM22871, GM03123 and GM05659; 10–16 cells/condition from GM18453) was determined using NIH ImageJ2 software. GraphPad Prism 10.6 was used for statistical analysis. The mean and standard deviation (SD) is shown. One-way ANOVA followed by Dunnett’s post-hoc test was used to determine statistical significance. * p,0.05, ** *p* < 0.01, *** *p* < 0.001. **** *p* < 0.0001. Bar is 10 μm. **(c-d)** NPC1 patient (GM22871, GM03123) and control fibroblasts (GM05659) were grown in 10% LPDS for 48 h, followed by lipid-loading in 10% FCS ± 50 μM compound **3**, **6** and **10** for 2 h as indicated. Cell lysates were prepared and Rab7-GTP pulldown assays were performed as described (see Methods for details). The amounts of active (Rab7-GTP) in pulldowns**,** total Rab7 and β-actin in whole cell lysates were determined by western blotting and representative images from two independent pulldown assays with lysates from NPC1 patient fibroblasts (GM22871, GM03123) are shown. For NPC1 patient fibroblasts, three independent experiments ± 50 μM compound **10** and for control fibroblasts, two independent experiments ± 50 μM compound **3**, **6** and **10** were quantified using ImageJ2 software. The mean and standard deviation (SD) is shown. GraphPad Prism 10.6 was used for statistical analysis (unpaired Student’s t-test). * p,0.05, **** *p* < 0.0001. **(e–f)** NPC1 mutant M12 cells were grown on coverslips and transfected with scrambled siRNA or siRNA targeting Rab7a. After 48 h lipid starvation in LPDS-containing media, cells were grown in 10% FCS ± 10 mM compound **10** for 24 h. Cells were fixed and free cholesterol was stained with GST-PFO (see Methods for details). Representative images of starved cells (LPDS) and cells incubated with DMSO or 10 mM compound **10** are shown. Multiple images from each condition were captured at identical settings with fixed intensities below their saturation. Fluorescence intensity (25–30 cells/condition from 5–7 images) was determined using NIH ImageJ2 software. GraphPad Prism 10.6 was used for statistical analysis. The mean and standard deviation (SD) is shown. One-way ANOVA followed by Dunnett’s post-hoc test was used to determine statistical significance. **** *p* < 0.0001. Bar is 10 μm. **(g)** Western blot analysis of Rab7 and β-actin from cell lysates of M12 cells transfected ± scrambled or Rab7 siRNA. ImageJ2 software was used for quantitative analysis of band intensity (mean ± SD).

The potency of drug candidates to overcome cholesterol accumulation was then analyzed in fibroblasts from three NPC1 patients, each carrying different NPC1 gene mutations (GM22871: 1920delG, 129 bp insertion, IVS9-1009G > A; GM03123: splice site mutation 1947 + 5G > C and missense 709 C > T (P237T), second allele 3182 T > C (I1061T); GM18453: homozygous 3182 T > C (I1061T)). Wildtype GM05659 fibroblasts served as controls. Fibroblasts were starved in 10% LPDS for 48 h followed by cholesterol loading with 10% FCS in the presence or absence of DMSO (0.1%) or 10 μM (**9**) or 50 μM (**3**, **6**, **10**) lead compounds for additional 24 h (Fig. 4a-b). Consistent with the results from experiments using NPC1 mutant CHO cells (Fig. 2a-d), following lipid starvation, FCS-mediated cholesterol loading increased filipin staining intensity (~ 1.8—threefold) in all three NPC patient fibroblast lines. Furthermore, supplementation with compound **3**, **6**, **9** and **10** significantly reduced filipin staining intensity in FCS-incubated GM22287 (3.8—fivefold), GM03123 (1.6—twofold) and GM18453 fibroblasts (2—3.9-fold) compared to FCS-loaded cells (Fig. 4b). In contrast, cholesterol staining intensity in FCS-loaded control fibroblasts was not significantly altered upon incubation with the four drug candidates. Taken together, compounds **3**, **6**, **9** and **10** can activate cholesterol export pathways in fibroblasts lacking NPC1 or expressing the misfolded NPC1^I1061T^ mutant protein.

To examine the ability of compounds to upregulate Rab7 activity in NPC patient fibroblasts, we determined Rab7-GTP levels in GM22871, GM03123 and control (GM05659) fibroblasts grown in LPDS for 48 h, followed by 2 h lipid-loading with 10% FCS ± 50 μM compound **3**, **6** and **10**. Lysates were prepared, subjected to pulldown assays as described above and analyzed by western blotting (Fig. 4c-d). Rab7 expression levels were elevated by ~ 20–30% in NPC1 patient fibroblasts compared to their wildtype counterparts (Supplementary Fig. S4b), which could reflect increased amounts of membrane-associated Rab7 due to late endosomal cholesterol accumulation that has been observed previously [17, 18]. In contrast, TBC1D15 levels decreased in both NPC1 patient fibroblast lines compared to control (Supplementary Fig. S4b). Alike results obtained from drug-treated M12 cells (see Fig. 3c-d), compounds **3**, **6** and **10** elevated Rab7-GTP levels in GM22871 and GM03123 fibroblasts (Fig. 4c), with increased Rab7 activity being significant for compound **10** (see quantification in Fig. 4d). Critically, compounds **3**, **6** and **10** did not significantly elevate Rab7-GTP levels in wildtype GM05659 fibroblasts, which correlates with their lack of impact on filipin staining intensity in these cells (see Fig. 4b). Hence, the ability of compounds **3**, **6** and **10** to activate Rab7-GTP levels is likely responsible for overcoming the accumulated cholesterol phenotype of NPC1 mutant fibroblasts.

### Rab7 Depletion Hinders Drug Candidates from Reducing Cholesterol Accumulation in NPC1 Mutant Cells

To further validate that drug candidates reduced cholesterol accumulation in NPC1 mutant cells in a Rab7-dependent manner, M12 cells were transfected with siRNA targeting Rab7a. Cells transfected with scrambled siRNA served as control. Then cells were starved in 10% LPDS for 48 h, followed by an incubation with 10% FCS ± 50 μM compound **10** or DMSO for additional 24 h. Cells were fixed and cholesterol was stained using GST-PFO (Fig. 4e-f). Western blot analysis of duplicate samples revealed ~ 80% Rab7 knockdown efficiency (Fig. 4g). As described above (see Figs. 2 and 4a), compound **10** strongly reduced cholesterol accumulation in scramble siRNA expressing M12 cells (~ 1.9-fold). In striking contrast, compound **10** did not decrease cholesterol build-up in Rab7-depleted M12 cells, suggesting that its ability to elevate Rab7-GTP levels is the underlying mechanism promoting cholesterol removal in NPC1 mutant cells.

### Rab7 Overexpression and Drug Candidates Reduce Cholesterol Accumulation in Neuronal SH-SY5Y Cells

**Fig. 5:**
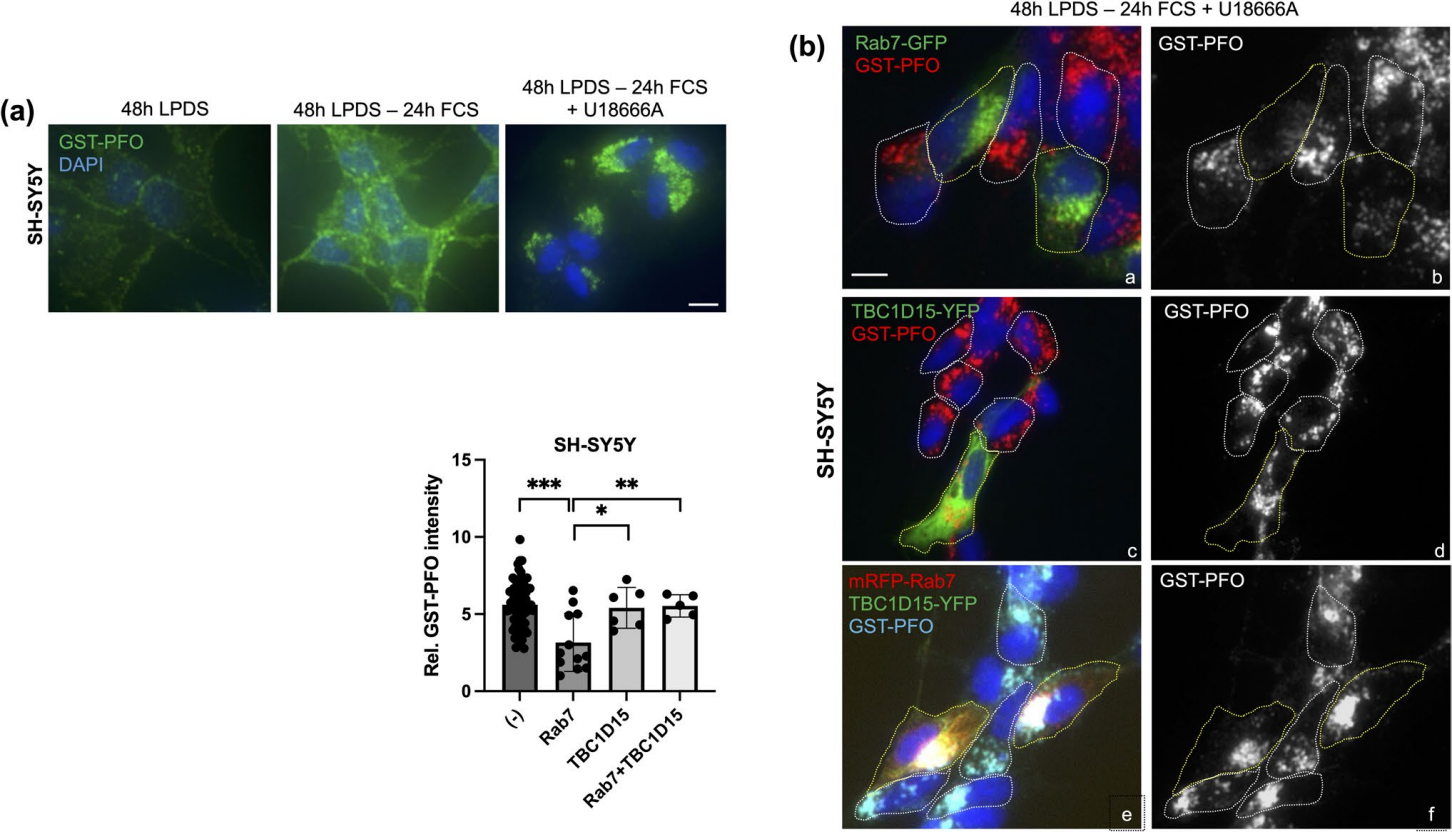

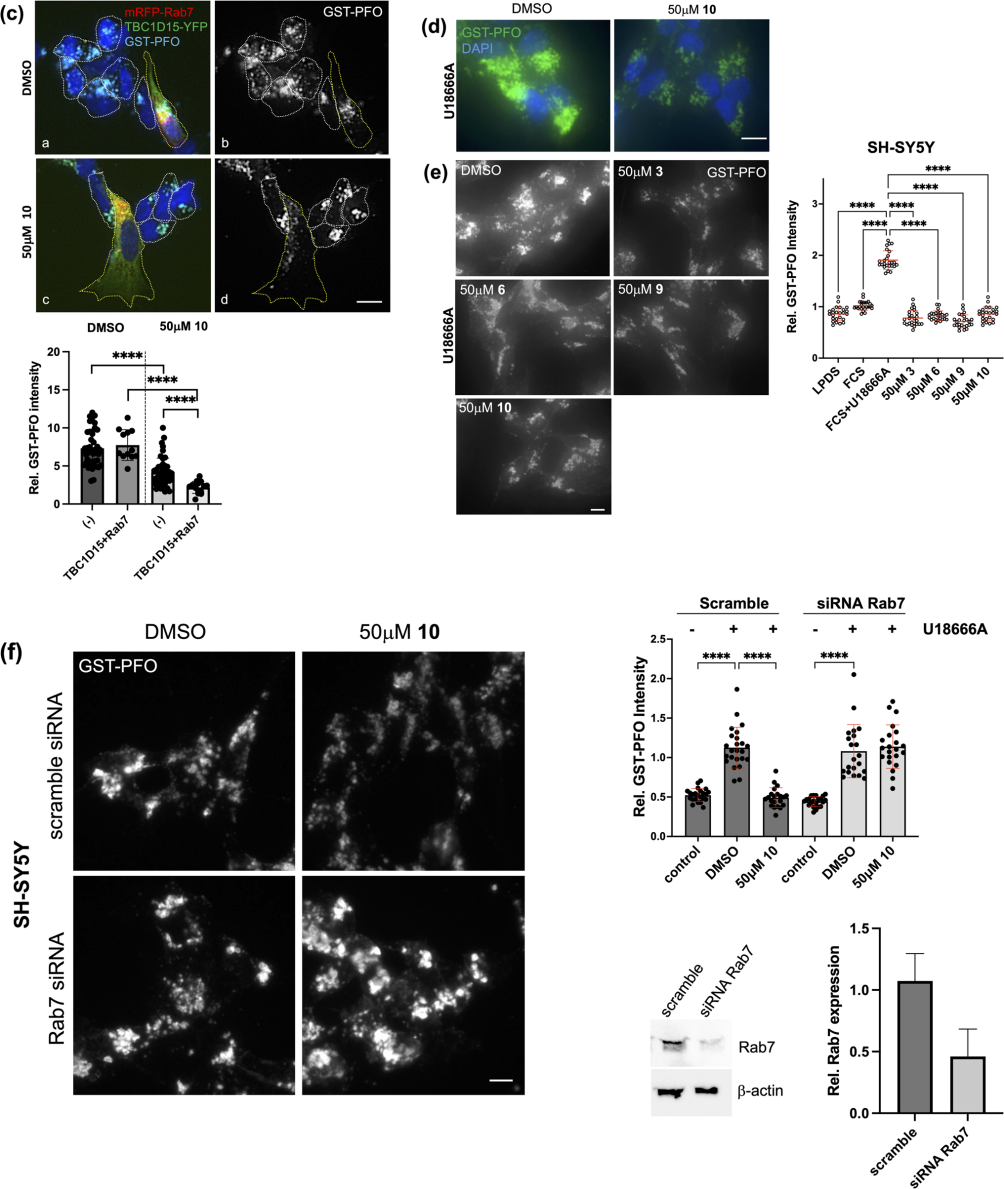

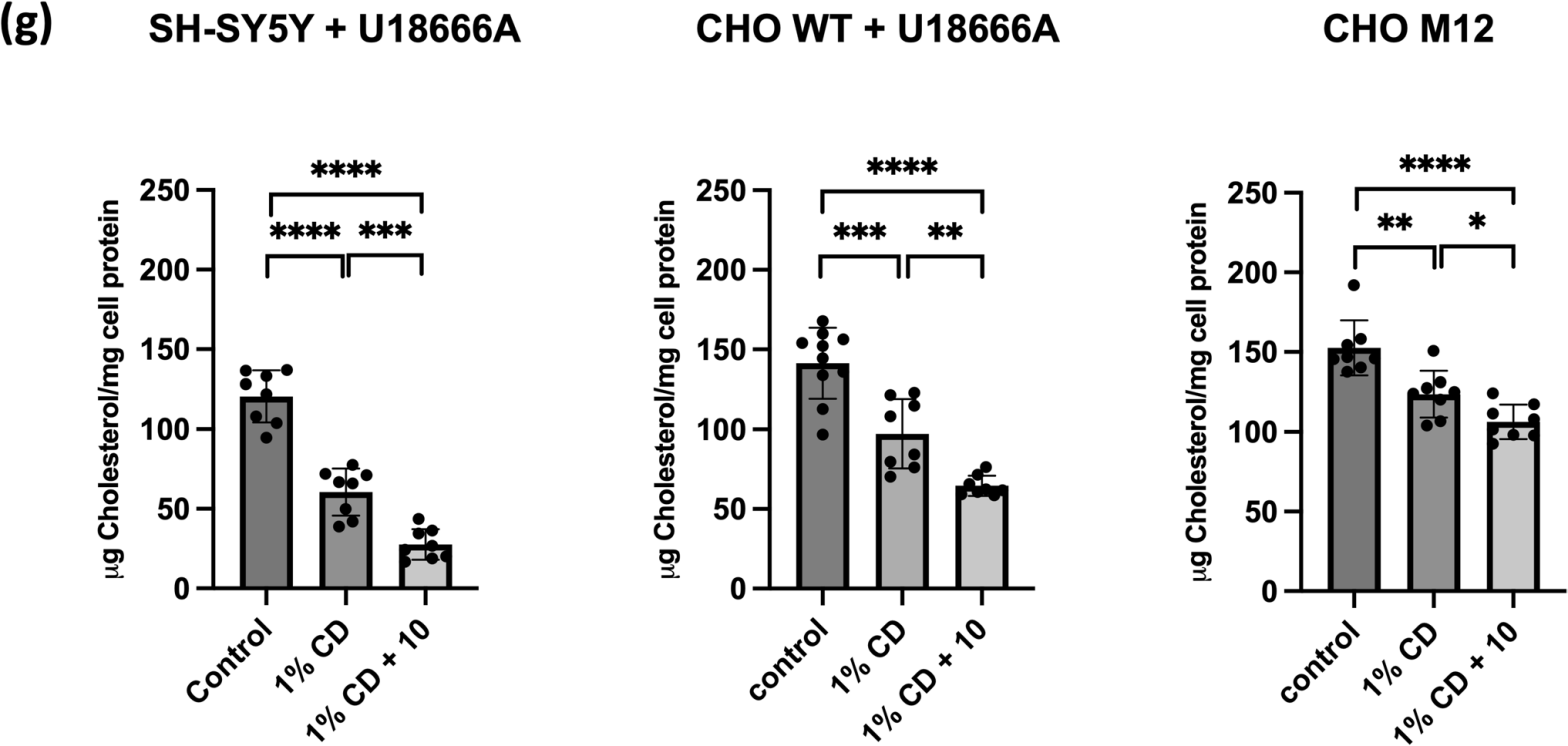
Rab7 overexpression and drug candidates reduce cholesterol accumulation in the SH-SY5Y neuroblastoma cell line. **(a)** SH-SY5Y cells on coverslips were grown in LPDS-containing media for 48 h. After lipid depletion, cells were grown in media with 10% FCS ± U18666A (2 μg/ml) for 24 h. Cells were fixed and free cholesterol was stained with GST-PFO (green) (see Methods for details). Nuclei were stained with DAPI (blue). Bar is 10 μm. **(b)** SH-SY5Y cells were transfected with Rab7-GFP (green; panel a-b), TBC1D15-YFP (green, panel c-d), or mRFP-Rab7 (red) together with TBC1D15-YFP (green, panel e–f) as indicated. After transfection, cells were starved in LPDS-containing media and then incubated in media with 10% FCS and U18666A (2 μg/ml) for 24 h. Cells were fixed and free cholesterol was stained with GST-PFO (red in panel a and c, cyan in panel e) as described. Black and white images from the GST-PFO staining are shown (panels b, d and e). Multiple images from each transfection were captured at identical settings with fixed intensities below their saturation. Fluorescence intensity of GST-PFO in non-transfected (n = 70) and transfected cells (n = 5–12) was determined using NIH ImageJ2 software. **(c)** SH-SY5Y cells were co-transfected with mRFP-Rab7 (red) and TBC1D15-YFP (green) and incubated as described in (b) ± DMSO (top panels) or 50 μM compound **10** (bottom panels) for 24 h as indicated. Cells were fixed and free cholesterol was stained with GST-PFO (cyan). Black and white images from the GST-PFO staining are shown (right panels). Fluorescence intensity of GST-PFO in non-transfected (n = 46) and transfected cells (n = 12–15) was determined using NIH ImageJ2 software. **(b-c)** For better comparison of cholesterol (GST-PFO) staining, the outline of non-transfected (white) and transfected (yellow) cells is indicated. GraphPad Prism 10.6 was used for statistical analysis. The mean and standard deviation (SD) is shown. Unpaired Student’s t-test was used to determine statistical significance (B, C). * *p* < 0.05, ** *p* < 0.01, *** *p* < 0.001, **** *p* < 0.0001. Bar is 10 μm. **(d)** SH-SY5Y cells were incubated as described in (A) ± DMSO or 50 μM compound **3**, **6**, **9** and **10** for 24 h as indicated. Cells were fixed and free cholesterol was stained with GST-PFO (green). Nuclei were stained with DAPI (blue). **(e)** Black and white images from the GST-PFO staining of SH-SY5Y cells incubated as described in (D) with DMSO or 50 μM compound **3**, **6**, **9** and **10** are shown. GraphPad Prism 10.6 was used for statistical analysis. The mean and standard deviation (SD) is shown. One-way ANOVA followed by Dunnett’s post-hoc test was used to determine statistical significance. **** *p* < 0.0001. Bar is 10 μm. **(f)** SH-SY5Y cells were grown on coverslips and transfected with scrambled siRNA or siRNA targeting Rab7a. After 48 h lipid starvation in LPDS-containing media, cells were grown in 10% FCS ± DMSO or 50 μM compound **10** for 24 h. Cells were fixed and free cholesterol was stained with GST-PFO. 5–8 images from each transfection were captured at identical settings with fixed intensities below their saturation and representative images are shown. **(e–f)** Fluorescence intensity of GST-PFO from 25 (E) or 21–24 (F) cells/condition was determined using NIH ImageJ2 software. GraphPad Prism 10.6 was used for statistical analysis. The mean and standard deviation (SD) is shown. One-way ANOVA followed by Dunnett’s post-hoc test was used to determine statistical significance. **** *p* < 0.0001. Bar is 10 μm. A representative western blot of Rab7 and β-actin from cell lysates of SH-SY5Y cells transfected ± scrambled or Rab7 siRNA is shown (n = 3). ImageJ2 software was used for quantitative analysis of band intensity (mean ± SD). **(g)** SH-SY5Y, CHO-WT cells and CHO M12 cells were starved for 48 h in LPDS-containing media, then incubated in 10% FCS-containing media and U18666A (2 μg/ml) for 24 h. Cells were then pre-incubated ± 50 μM compound **10** for 2 h as indicated, followed by 60 min treatment in serum-free media containing 1% 2-hydroxypropyl-β-cyclodextrin (HPβCD). The media was removed and cell lysates were prepared. Lipids were extracted and cellular cholesterol was measured using the Amplex Red Cholesterol Assay kit as described (see Methods for details).

To date, the ability of upregulated Rab7 activity to bypass NPC1 deficiency and activate alternative cholesterol export pathways has been demonstrated only in NPC1 patient fibroblasts and NPC1 mutant CHO cells [12, 15, 16]. Given the clinical relevance of neurons in NPC disease, we performed experiments in the human neuroblastoma SH-SY5Y cell line, which displays many biochemical and functional properties of human neurons and has been widely used as a model for neurodegenerative diseases. In fact, pharmacological NPC1 inhibition applying the amphipathic steroid U18666A in SH-SY5Y cells previously served as a model for NPC disease [57, 58]. Here, and consistent with these earlier reports and alike other NPC1 mutant models, 24 h treatment of SH-SY5Y cells with U18666A (2 μg/ml) lead to a substantial increase in cholesterol staining intensity in the perinuclear LE/Lys compartment (Fig. 5a, for quantification see Fig. 5d). Concomitantly, as pharmacological NPC1 inhibition leads to a reduction in esterified cholesterol due to the lack of cholesterol transport to the ER, this likely contributes to the moderate increase in total cellular cholesterol levels determined biochemically when compared to controls (Supplementary Fig. S4c) [46, 47, 59, 60].

To determine if Rab7 overexpression could rescue the NPC1 phenotype in human neuroblastoma, SH-SY5Y cells were transfected with GFP-tagged Rab7. Indeed, transient overexpression of Rab7 reduced U18666A-induced cholesterol accumulation by ~ 45% (Fig. 5b), which is similar to previous findings in fibroblasts [12, 15, 16]. This highlights the ability of ectopic Rab7 overexpression to overcome pharmacological NPC1 inhibition in SH-SY5Y cells. To assess if TBC1D15 overexpression could inhibit Rab7-mediated rescue of the NPC1-mutant-like phenotype, SH-SY5Y cells were co-transfected with mRFP-tagged Rab7 and YFP-tagged TBC1D15. While TBC1D15 overexpression alone did not impact on U18666A-induced cholesterol accumulation, ectopically expressed Rab7 failed to rescue cholesterol export from LE/Lys when co-expressed with TBC1D15. Yet, this was not observed when U18666A-treated SH-SY5Y cells co-expressing mRF-Rab7a and TBC1D15-YFP were incubated with 50 μM compound **10** (Fig. 5c). Hence, this indicates that compound **10** can overcome TBC1D15-mediated Rab7 downregulation to reduce cholesterol accumulation in cells lacking NPC1 activity (Fig. 5b-c).

Given the presence of Rab7-inducible and NPC1-independent cholesterol transport pathways capable of bypassing NPC1 inhibition in SH-SY5Y cells, we then examined the potential of drug candidates **3**, **6**, **9** and **10** to overcome pharmacological NPC1 inhibition in these cells (Fig. 5d-e). U18666A treatment of SH-SY5Y cells resulted in an almost twofold increase in GST-PFO staining intensity. Strikingly, addition of 50 μM compound **3**, **6**, **9** or **10** for 24 h reduced GST-PFO staining intensity by 2.3—2.8-fold in U18666A-treated SH-SY5Y cells, respectively. Furthermore, transfection of SH-SY5Y cells with siRNA targeting Rab7a (~ 50–76% knockdown efficiency), followed by an incubation with 10% FCS and U18666A (2 μg/ml) ± 50 μM compound **10**, strongly reduced the ability of compound **10** to decrease cholesterol accummulation in Rab7-depleted SH-SY5Y cells (Fig. 5f). This was similar to the data generated in M12 cells (see Fig. 4e-g), further supporting evidence that compound **10** promotes cholesterol removal in a Rab7-dependent manner in NPC1 mutant cells.

To assess if drug candidates render late endosomal/lysosomal cholesterol more accessible for cholesterol-depleting agents, U18666A-treated SH-SY5Y were incubated ± 50 μM compound **10** cells for 2 h, followed by 60 min with 1% HPβCD (Fig. 5g). Treatment of cholesterol-loaded SH-SY5Y cells with HPβCD effectively reduced cellular cholesterol levels by ~ 47%. Impressively, pre-incubation for 2 h with 50 μM compound **10** further augmented HPβCD-inducible cholesterol removal to ~ 62%. Likewise, compound **10** improved the ability of HPβCD to extract cholesterol from U18666A-treated CHO-WT cells and M12 cells by ~ 23% and ~ 11%, respectively (Fig. 5g). These results suggest that there is therapeutic potential for combining HPβCD with Rab7 activators for the treatment of NPC1 disease.

### Drug Candidates Reduce U18666A-induced Cholesterol Accumulation in Differentiated SH-SY5Y Cells and Neuronal Organoids

**Fig. 6:**
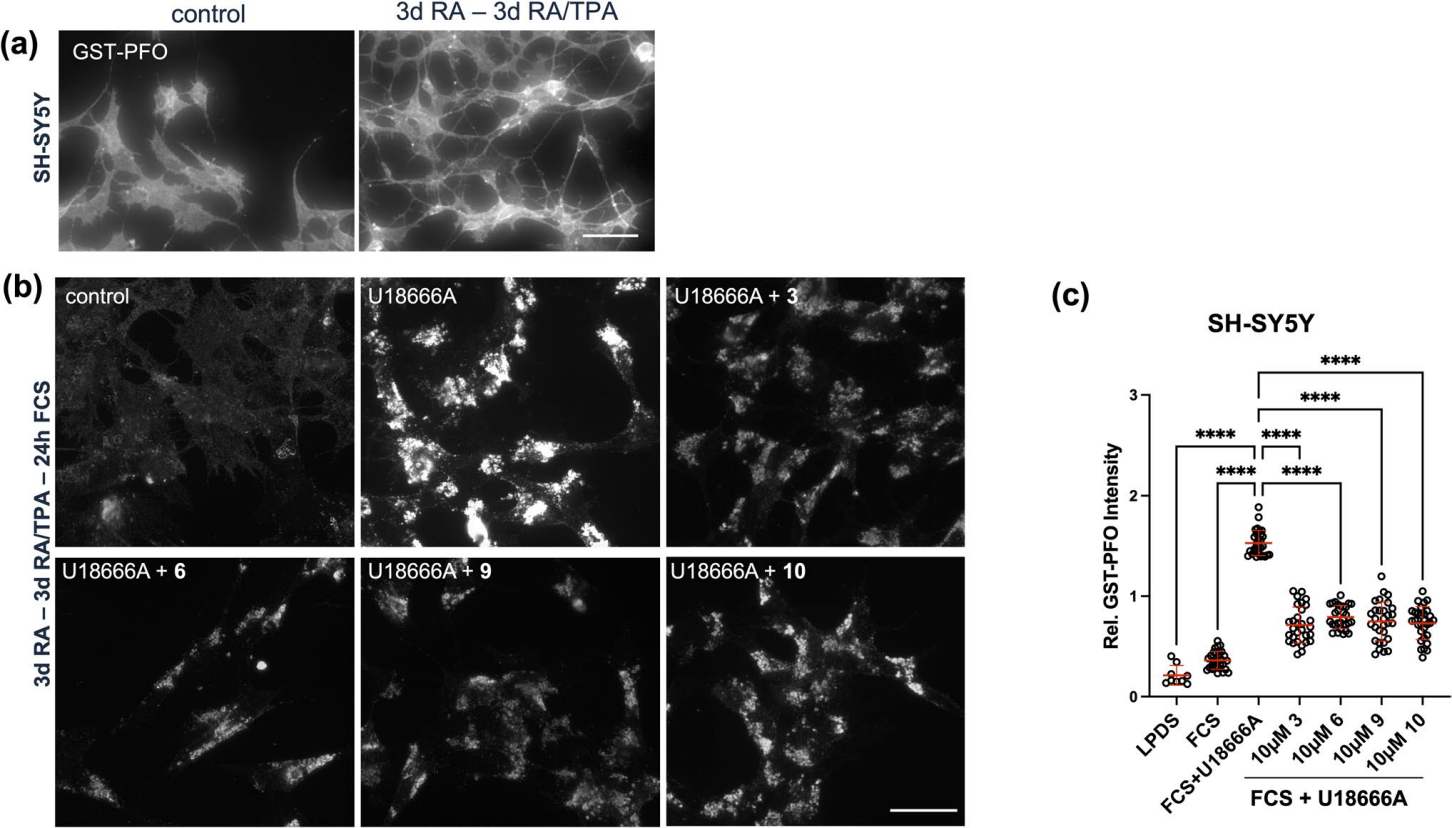

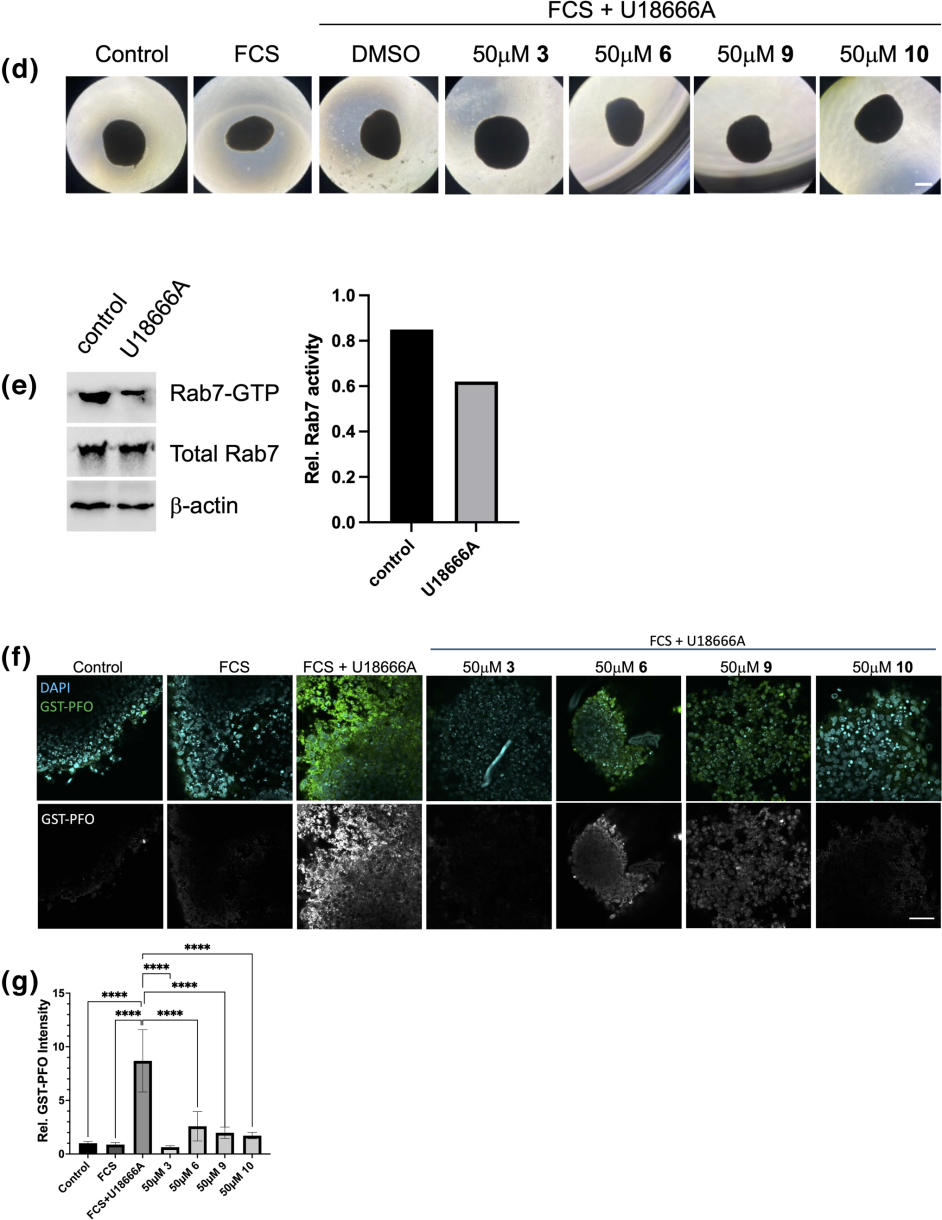
Drug candidates reduce U18666A-induced cholesterol accumulation in differentiated SH-SY5Y cells and 3D-brain organoids. **(a-b)** SH-SY5Y cells on coverslips were treated for 3 days with 10 μM RA, followed by 3 days in 10 μM RA and 81 nM TPA (see Methods for details). Cells were grown in 10% LPDS for 48 h, followed by 24 h lipid loading in 10% FCS in the presence of U18666A (2 μg/ml) ± 10 μM compounds **3**, **6**, **9** or **10** as indicated. Cells were fixed and free cholesterol was stained with GST-PFO. Bar is 50 μm. **(c)** 5–8 images from each transfection were captured at identical settings with fixed intensities below their saturation and representative images are shown. Fluorescence intensity of GST-PFO of 30 cells/condition (n = 9 for LPDS) from 5–8 images/condition was determined using NIH ImageJ2 software. GraphPad Prism 10.6 was used for statistical analysis. The mean and standard deviation (SD) is shown. One-way ANOVA followed by Dunnett’s post-hoc test was used to determine statistical significance. **** *p* < 0.0001. **(d-e)** Human dorsal brain organoids were generated from human embryonic stem cells (hESC, wildtype) (see Methods for details). After at least 45 days maturation, 3D-brain organoids were treated 48 h with 10% FCS ± U18666A (2 μg/ml). Lysates from ~ 10–12 organoids were prepared and subjected to RILP-C33-GST pulldown assays as described and analyzed by western blotting to determine Rab7 activity. Bar is 2 mm (A). **(f-g)** 3D-brain organoids were treated 48 h with 10% FCS ± U18666A as above in the presence or absence of 50 μM of compounds **3**, **6**, **9** and **10**. Organoids were fixed and cholesterol was stained with anti-GST-PFO (green) together with DAPI (cyan, nuclei). Black and white images from the GST-PFO staining are shown in the bottom panels. For each treatment, 5–6 images from organoids were captured at identical settings with fixed intensities below their saturation and representative images are shown. Fluorescence intensity of GST-PFO of 4–8 tissue sections from 2–3 representative images per condition was determined using NIH ImageJ2 software. GraphPad Prism 10.6 was used for statistical analysis. The mean and standard deviation (SD) is shown. One-way ANOVA followed by Dunnett’s post-hoc test was used to determine statistical significance. **** *p* < 0.0001. Bar is 100 μm (C).

SH-SY5Y cells can be differentiated into a more neuron-like phenotype characterized by neurite outgrowth following RA and phorbol ester (TPA) treatment [61]. To examine the cholesterol-removing capacity of drug candidates in a neuron-like cell culture model, SH-SY5Y cells were treated for 3 days with 10 μM RA, followed by 3 days in 10 μM RA and 81 nM TPA as described [37] (Fig. 6a). Cells were grown in 10% LPDS for 48 h, followed by 24 h lipid loading in 10% FCS in the presence of U18666A (2 μg/ml) ± 10 μM compounds **3**, **6**, **9** or **10**, respectively. As expected, pharmacological inhibition of NPC1 increased cholesterol accumulation 4.2-fold (Fig. 6b-c). Most excitingly, incubation with compounds **3**, **6**, **9** and **10** reduced late endosomal cholesterol build-up by 1.9—2.1-fold. These findings point at the potential of elevated Rab7 activity to overcome NPC1 deficiency in neuronal cells.

Finally, to exame whether the four lead compounds could penetrate brain-like tissue and overcome NPC1 inhibition in 3D-environments, we prepared brain organoids from human wildtype embryonic stem cells (hESC) as described [44]. Brain organoids are brain-like tissues that self-organize *in vitro* from human pluripotent stem cells following human brain development stages and recapitulating stage-specific gene expression patterns. They comprise multiple neural lineages, including neurons, neuronal progenitor cells, and glia cells (astrocytes, oligodendrocytes), and exhibit native developmental cytoarchitecture and functional network activity reminiscent of the developing human brain, thereby providing a more human-relevant model for brain disease research compared to animal models or 2D *in vitro* systems [44].

Human dorsal brain organoids were generated from pluripotent wildtype stem cells (see Methods for details). After at least 45 days of maturation, we first examined the cholesterol-sensitive activity of Rab7 and treated 3D-brain organoids 48 h with 10% FCS ± U18666A (2 μg/ml). Lysates were prepared and subjected to RILP-C33-GST pulldown assays as described above and analyzed by western blotting (Fig. 6d-e). In line with late endosomal cholesterol accumulation interfering with Rab7 activity in cell-based models [12, 17, 18], U18666A treatment of brain-like organoids from wildtype hESC resulted in a ~ 25% reduction of Rab7-GTP levels and caused a significant cholesterol accumulation (Fig. 6f; for quantification see Fig. 6g). Most impressively, addition of 50 μM lead compounds **3**,** 6**, **9** and **10** to U18666A-treated organoids for 24 h strongly reduced cholesterol staining intensity (Fig. 6f-g). These findings indicate that the lead compounds can successfully penetrate tissue-like environments to target TBC1D15 and upregulate Rab7-dependent cholesterol transport pathways.

## Discussion

We previously demonstrated that elevating Rab7-GTP levels upon Rab7-GAP TBC1D15 gene depletion enabled activation of cholesterol export routes from LE/Lys, reducing cholesterol accumulation in NPC1 mutant cells [12]. Here we describe the identification of four drug candidates with potentially high TBC1D15 binding affinity that raise Rab7-GTP levels and promote cholesterol export from LE/Lys in NPC1 mutant CHO cells, NPC1 patient fibroblasts as well as differentiated SH-SY5Y neuronal cells and 3D-brain organoids treated with a pharmacological NPC1 inhibitor. Moreover, drug candidates potentiated the ability of HPβCD to reduce cellular cholesterol accumulation. Hence, advancing small molecules that upregulate Rab7-GTPase activity could provide opportunities to activate NPC1-independent pathways that can overcome and bypass cholesterol transport defects in NPC1 mutant cells.

Two of the currently approved medications to treat NPC disease represent disease-modifying therapeutics that delay neurodegeneration and disease progression rather than targeting the inability of NPC mutants to export cholesterol from LE/Lys. Miglustat is a small iminosugar that inhibits glucosylceramide synthase, thereby reducing neuronal glycosphingolipid accumulation and delaying the onset of neuronal dysfunction [3, 62]. However, miglustat lacked potency to reduce cholesterol accumulation in NPC mutant cells, in induced pluripotent stem cells (iPSC) neurons from NPC1 patients, or in an NPC1 animal model [63–65]. Additionally, the modified amino acid *N*-acetyl-L-leucine (NALL) improves energy metabolism, thereby benefiting mitochondrial and lysosomal function. This also includes a reduction in late endosomal/lysosomal cholesterol accumulation, which contributes to slow overall disease progression and neurodegeneration [4]. Arimoclomol on the other hand activates transcription factors that upregulate genes responsible for improved lysosomal function and amplified heat shock protein (hsp) expression. The latter enables misfolded NPC1 mutants to escape degradation in the endoplasmic reticulum and to reach LE/Lys to reduce cholesterol accumulation [66]. While most existing hsp inducers can provoke cell toxicity, Arimoclomol demonstrated a safety profile suitable for chronic use [67].

Other therapeutic efforts that target the build-up of cholesterol in LE/Lys of NPC mutants include histone deacetylase (HDAC) inhibitors such as Vorinostat. Like Arimocromol, HDAC inhibitors correct the trafficking defect of misfolded NPC1 mutants (e.g. I1061T) to restore cholesterol export from LE/Lys [5, 68]. The multiple HDAC isoforms and the limited selectivity of HDAC inhibitors, together with their antiproliferative effects [69], complicate further drug development. Alternatively, HPβCD is a cyclic oligosaccharide with a hydrophobic center that can directly bind and export late endosomal cholesterol [7]. In NPC1-deficient mice and several phase 1/2a trials, both intrathecal and intravenous administration [7–9] of HPβCD reduced cholesterol accumulation with beneficial effects for neurological symptoms, and improved liver dysfunction [7, 70]. Yet, associated risks of neurotoxicities exist, including hearing loss [7, 9, 71].

Furthermore, exploration of NPC1-independent cholesterol export routes has the potential to correct NPC disease. This includes LBPA, which promotes export of late endosomal cholesterol through pathways that can bypass the NPC1 protein [10]. Most relevant for this study, gain-of-function experiments, using ectopic overexpression of wildtype or constitutively active Rab mutants (Rab4, 7, 8, 9) or a Rab7 guanine nucleotide exchange factor, reduced cholesterol accumulation in NPC1 mutant cells and patient fibroblasts [12, 15, 16] (reviewed in [72]).

Genetic upregulation of Rab activities remains challenging to develop therapeutically, as transgenes may trigger additional and undesired biological activities. To date, only transgenic overexpression of Rab9 has been analyzed in an NPC mutant mouse model [73]. Indeed, this approach extended the lifespan of NPC1-deficient animals and reduced ganglioside storage in the cerebral cortex and hippocampus. Yet, filipin staining did not reveal any reduction in cholesterol accumulation in brain sections of these NPC mutant mice overexpressing Rab9, indicating limited or differential activities of the Rab9 transgene that may not impact on cholesterol export from LE/Lys *in vivo* [73].

Based on our genetic approaches [12], we aimed to pharmacologically upregulate endogenous Rab7-GTP levels to activate cholesterol export routes that can overcome NPC1 deficiency. Currently, ML-098 is the sole pharmacological drug molecule available for Rab7 activation. ML-098 interacts with Rab7 at an allosteric binding site that increases the affinity of Rab7 for GTP nucleotides. However, while ML-098 shows a preference for Rab7, it also interacts with several other members of the Ras GTPase superfamily, including Cdc42, Ras, Rab2a and Rac1, thus limiting its clinical use [74].

Rather than developing small molecules targeting the GTP-binding capacity of Rab7, we aimed to pharmacologically interfere with TBC1D15-mediated Rab7 inactivation. To achieve this, we used previously established in silico drug design approaches [22, 23]. We first modelled the 3D-structure of the human TBC1D15 GAP domain in complex with human Rab7-GTP, which aligned with the crystal structures of the Shark and Sus TBC1D15 GAP domains bound to human Rab7a [48]. We then identified druggable sites at the TBC1D15/Rab7 interface that would allow the discovery of drug candidates that can bind TBC1D15 and interfere with TBC1D15-mediated Rab7 inactivation. We next applied ligand-based in silico screening techniques and structure-based drug discovery approaches*,* using docking-based virtual screening of a compound library, to identify a panel of drug candidates with interaction patterns and estimated binding modes indicative of high binding affinity for TBC1D15. Indeed, four of the top 10 drug candidates increased Rab7-GTP levels in M12 and NPC1 patient fibroblasts, coinciding with reduced TBC1D15/Rab7-GTP complex formation and loss of TBC1D15 membrane association, altogether indicating reduced TBC1D15/Rab7 assembly. Most strikingly, IC_50_ values to lower cholesterol accumulation in NPC1 mutant cells for three of the four compounds (**3**, **6**, **10**) were in the mid-nanomolar to low-micromolar range. At these concentrations, cell viability, membrane leakage, and oxidate stress were not negatively affected. Despite these encouraging findings, it should be noted that biochemical assays using purified recombinant proteins will still be needed in future studies to validate that binding of the four drug candidates to TBC1D15 compromises Rab7-GTP hydrolysis.

The four lead compounds also showed potency to overcome cholesterol transport defects in NPC1 patient fibroblasts that lack the NPC1 protein or express the common NPC1^I1061T^ mutation, which is still functional but only present in low amounts in LE/Lys [5, 6]. The latter results are in line with recent findings that identified a direct interaction of active Rab7 with NPC1 [16]. This interaction remained unaffected by loss-of function NPC1 mutants that cannot bind cholesterol, cholesterol depletion or U18666A-induced LE/Lys-cholesterol accumulation and was proposed to rescue the cholesterol transport properties of the NPC1^I1061T^ mutant [16]. Hence, the drug attributes of second-generation molecules with improved potency could overcome NPC1 truncations and deficiency, and also benefit the most prevalent NPC1 missense mutation (NPC1^I1061T^).

Importantly, our studies revealed that upregulated Rab7 activity can bypass pharmacological NPC1 inhibition and activate alternative cholesterol export pathways in the human neuroblastoma SH-SY5Y cell line. In these cells, TBC1D15 overexpression inhibited Rab7-mediated rescue of the NPC1-mutant-like phenotype and all four drug candidates showed potency to overcome pharmacological NPC1 inhibition in differentiated SH-SY5Y cells, which represent a more neuron-like phenotype [57]. Moreover, the ability of drug candidate **10** to increase the efficacy of HPβCD in removing cellular cholesterol from U18666A-treated SH-SY5Y cells indicate a therapeutic potential for the combinatorial treatment of HPβCD and Rab7 activators.

We also examined the potential of drug candidates in more sophisticated *in vitro* human 3D-brain models that aim to recapitulate human brain development and neurological diseases [44]. In brain organoids derived from human pluripotent stem cells and treated with pharmacological NPC1 inhibitor, reduced Rab7-GTP levels correlated with a robust cholesterol accumulation. Most notably, incubation of U18666A-treated organoids with the four lead compounds strongly reduced cholesterol staining intensity, indicating that the lead compounds not only penetrate tissue-like environments, but also target TBC1D15 and upregulate Rab7-dependent cholesterol transport pathways to overcome NPC1 deficiency in models that simulate the human brain.

Several additional studies further point to Rab7-related activities as therapeutic targets to address NPC pathogenesis and other neurological disorders. Rab7 overexpression in mice was associated with increased autophagic activity [75], a mechanism proposed to lower cholesterol in NPC mutant cells [10, 76] that is possibly normalized in drug-treated NPC1 mutant cells in this study, as judged by the reduced amounts of autophagy markers. Along these lines, ML-098 improved autophagic fluxes, thereby reducing phenotypes associated with diabetic cardiomyopathy [77], pulmonary arterial hypertension [78] and osteonecrosis [79]. Alternatively, pharmacological targeting of other players in the LE/Lys compartment that are intimately linked to Rab7 activity, including the mammalian target of rapamycin and vacuolar-type ATPase, or capable of modifying Rab7 activity through phosphorylation, palmitoylation and ubiquitination, could offer therapeutic opportunities [80, 81]. As in this study, other examples of therapeutic targeting of the Rab7 GTP/GDP cycle in human disease exist. Depletion of the Rab7-GAP TBC1D5 elevated Rab7 activity to improve mitochondrial damage and alleviate neuronal dysfunction in a mouse model for Alzheimer’s disease [82]. In fruit flies expressing a pathogenic mutant to model Parkinson's disease, Rab7 overexpression increased clearance of α-synuclein aggregates and reduced cell death [83].

Of note, the TBC1D15/Rab7 complex and Rab7-GTP hydrolysis has been implicated in the regulation of membrane contact sites between LE/Lys and mitochondria and mitochondrial fission [84]*.* In fact, de-regulated inter-organelle communication between LE/Lys and mitochondria is a common phenomenon in neurological disorders and TBC1D15 depletion to elevate Rab7-GTP levels normalized the number of membrane contacts to correct amino acid dyshomeostasis in iPSC-derived dopaminergic neurons from patients with Parkin mutations [85]. On the other hand, elevated TBC1D15 expression to increase Rab7 hydrolysis reduced membrane contacts between LE/Lys and mitochondria to confer neuroprotective effects against neuronal injury induced by epileptic seizure [86]. TBC1D15-mediated Rab7 hydrolysis also improved mitochondrial dynamics in Parkinson’s disease patient-derived neurons carrying mutations in the glucocerebrosidase gene [87] and in models of Charcot-Marie-Tooth type-2B disease [88]. Given that NPC mutant models display de-regulated inter-organelle communication between LE/Lys and mitochondria, which correlates with compromised mitochondrial function in NPC disease [13, 89], the potential effects of the compounds identified in this study on mitochondrial function in NPC mutant models should be investigated in future studies.

## Conclusions

Taken together, the development of pharmacological molecules that elevate endogenous Rab7 activity via inhibition of TBC1D15-mediated Rab7 inactivation could be beneficial in NPC1 disease and other neurological disorders with endolysosomal dysfunction, and allow advances to better understand how Rab7- and TBC1D15-related activities contribute to Charcot-Marie-Tooth type 2B and lysosome regeneration in neurodegeneration [87, 90–94].

## Supplementary Information

Below is the link to the electronic supplementary material.

Supplementary file1 (DOCX 11838 KB)

## Data Availability

The datasets generated during and/or analysed during the current study are available from the corresponding author on reasonable request.
