# Supplementary material for: Small Molecules to Elevate Rab7-GTPase Activity and Lower Cholesterol Accumulation in Niemann-Pick Type C Disease
Source: Pharm Res. 2026 Mar 3;43(4):1009–31. doi: 10.1007/s11095-026-04058-8 (PMC13179269; doi:10.1007/s11095-026-04058-8)
Supplement: Supplementary file 1 — Supplementary file1 (DOCX 11838 KB) [file 11095_2026_4058_MOESM1_ESM.docx]

**Supplementary Information**

**Small molecules to elevate Rab7-GTPase activity and lower cholesterol accumulation in Niemann-Pick Type C disease (*Pharm Res*)**

Mai K.L. Nguyen, Maya R. Nikenich, Kim Seifert, Céline Pinkenburg, Felcia Lai, Hanna-Loisa Walther, Martje Hartmann, Aleksandra Szulc, Eric Sparkes, Shihui Chen, Ravi Wikramanayake, Marc Bernaus-Esqué, Yangjing Liu, Francesc Tebar, Michael Serwetnyk, Anna Wenninger, Christopher Patzke, Brian S.J. Blagg, Brandon L. Ashfeld, Paul W. Groundwater, David E. Hibbs, Andrew J. Hoy, Carles Rentero, Carlos Enrich, Ann-Na Cho, Jonathan Du, Thomas Grewal

*Corresponding author:

Thomas Grewal, School of Pharmacy, Faculty of Medicine and Health, University of Sydney, Sydney, NSW 2006, Australia; Tel +612 9351 8496; Email [thomas.grewal@sydney.edu.au](mailto:thomas.grewal@sydney.edu.au)


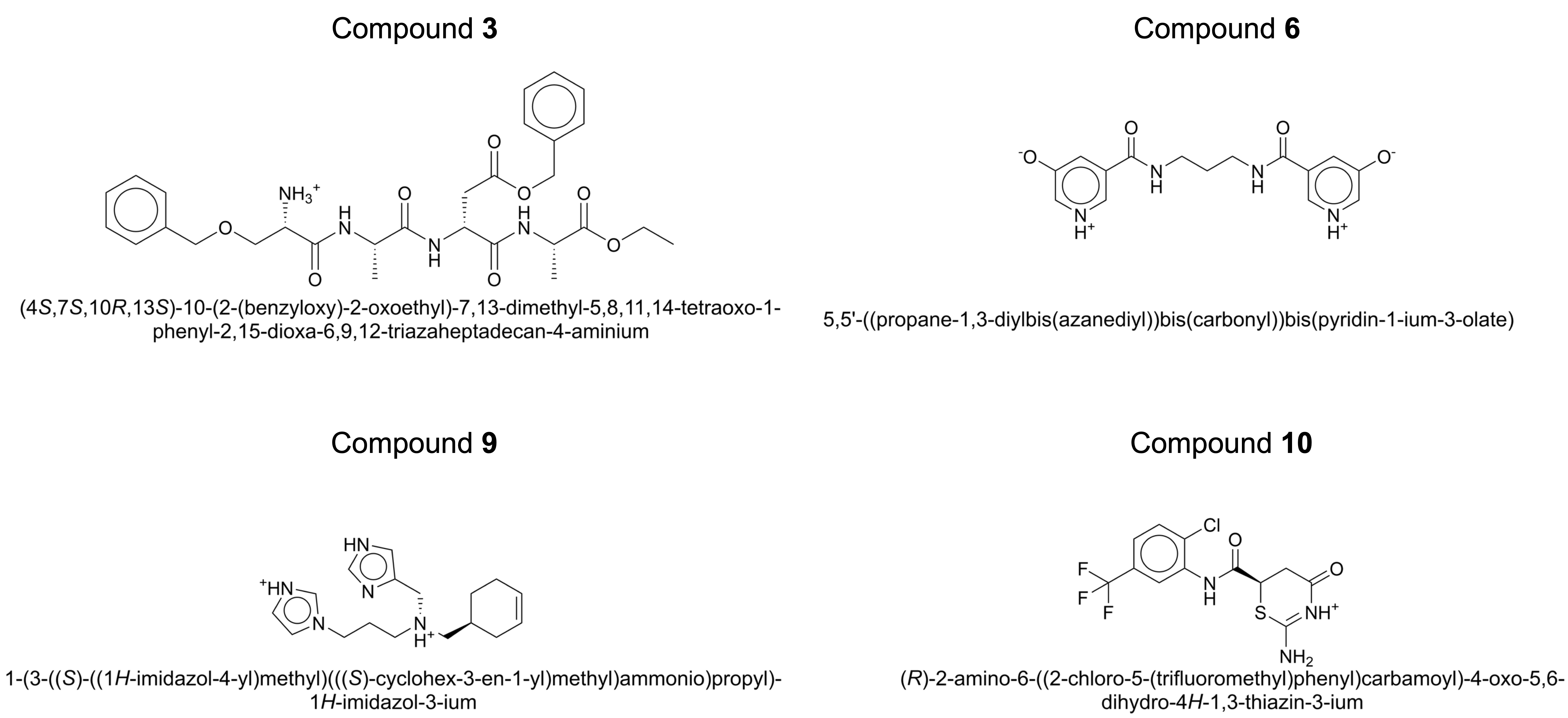


**Supplementary Fig. S1** Chemical structure of lead compounds. The structure and IUPAC nomenclature of the drug candidates **3** (CAS 1397056-96-0), **6** (CAS 400749-204), **9** (CAS 1011363-79-3) and **10** (CAS 431980-31-3) are shown (from ASINEX: BAS01402908, BAS05594113, ASN15408181 and BAS03450598).

**
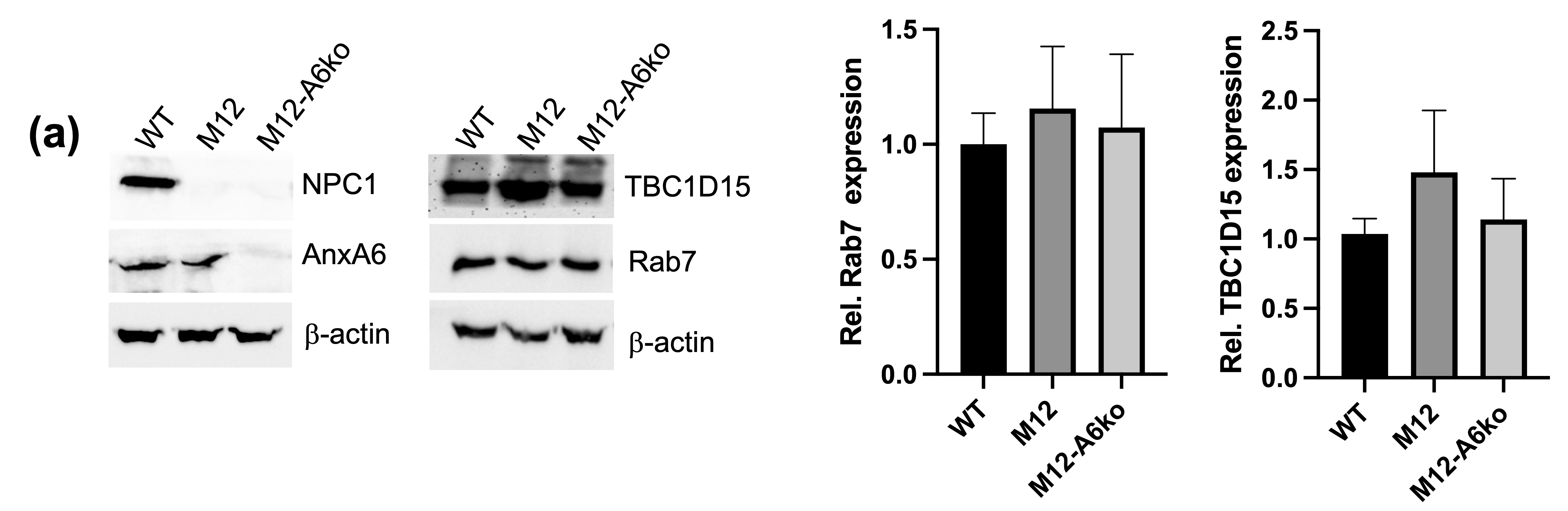
**

**
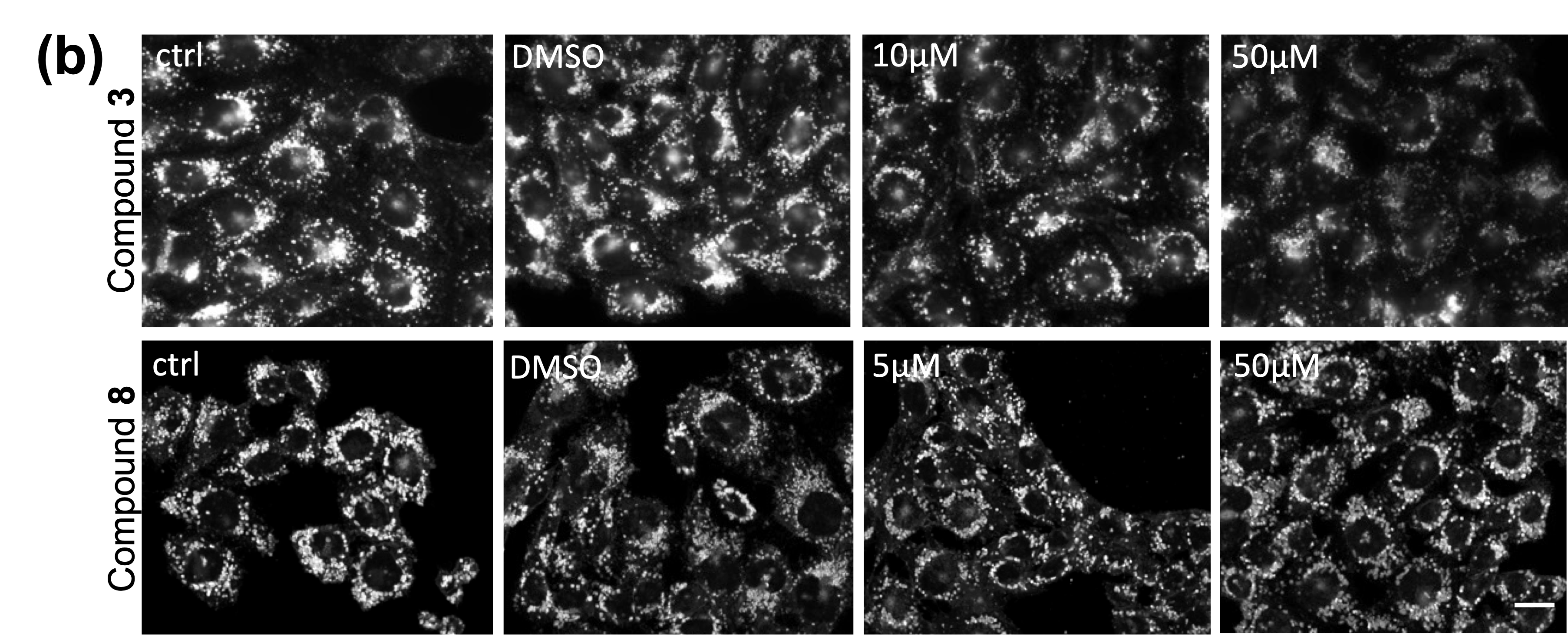
**

**
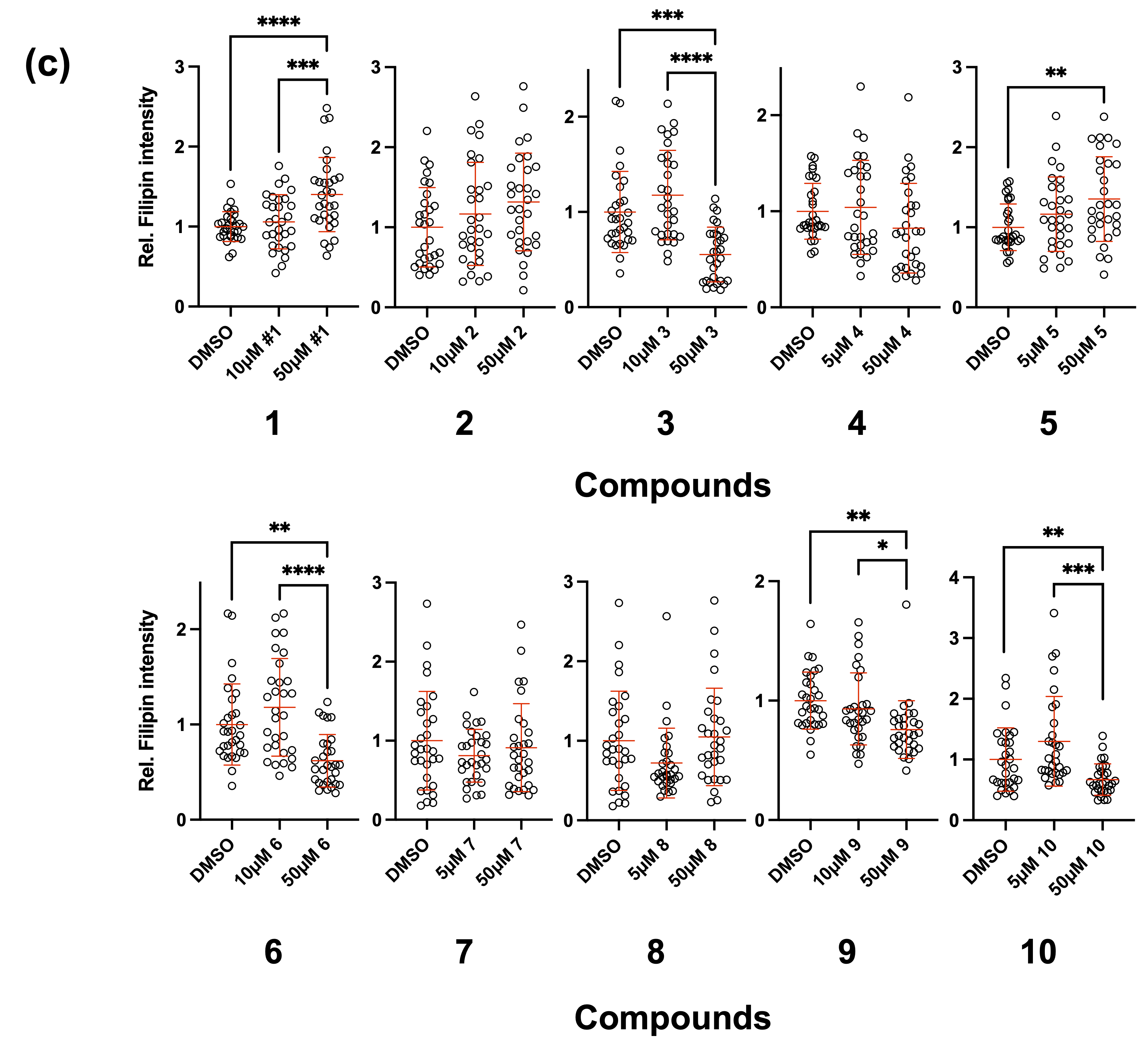
**

**Supplementary Fig. S2** Drug candidates 3, 6, 9 and 10 reduce cholesterol accumulation in NPC1 mutant M12 cells. **(a)** The amounts of NPC1, AnxA6, TBC1D15, Rab7 and β-actin levels in whole cell lysates from CHO wildtype (WT), NPC1 mutant (M12) and AnxA6-deficient NPC1 mutant M12 cells (M12-A6ko) were determined by western blotting. Expression levels of Rab7 and TBC1D15 levels were quantified using ImageJ2 software (mean ± SD). **(b)** CHO-M12 cells on coverslips were grown in 10% FCS ± 5, 10 or 50 μM compound **3**, **6**, **9** and **10** for 24 h. Cells were fixed and free cholesterol was stained with filipin as described (see Methods for details). Representative images of cells incubated without (ctrl) or with DMSO or 5, 10 or 50 μM compound **3** and **8** are shown. 5-8 images from each condition were captured at identical settings with fixed intensities below their saturation. Fluorescence intensity (30 cells/condition from 5-6 images) was determined using NIH ImageJ2 software. The mean and standard deviation (SD) is shown. GraphPad Prism 10.6 was used for statistical analysis (One-way ANOVA followed by Dunnett’s post-hoc test). * p<0.05, ** p<0.01, *** p<0.001, **** p <0.0001. Bar is 10 μm.

**
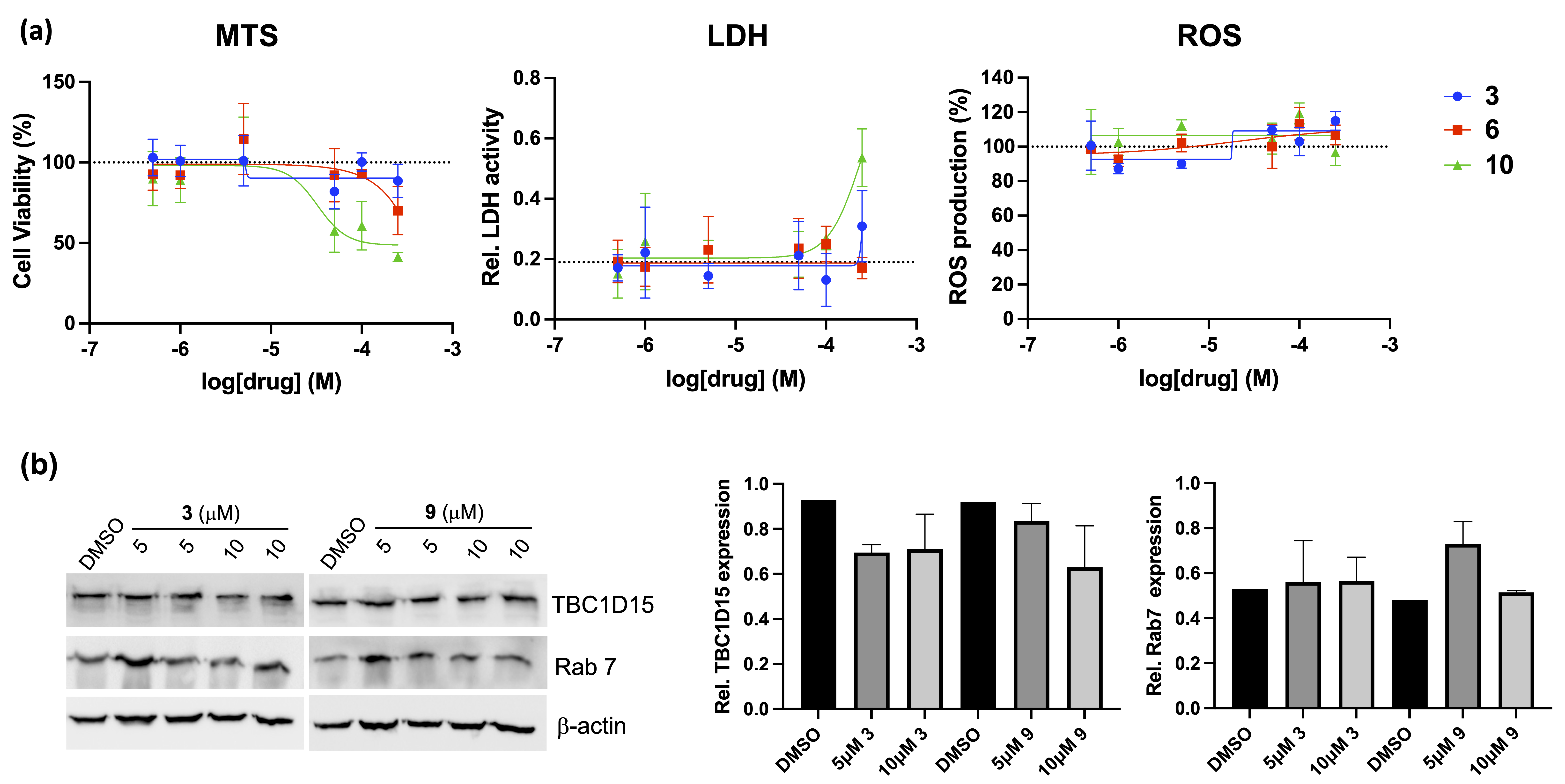
**

**
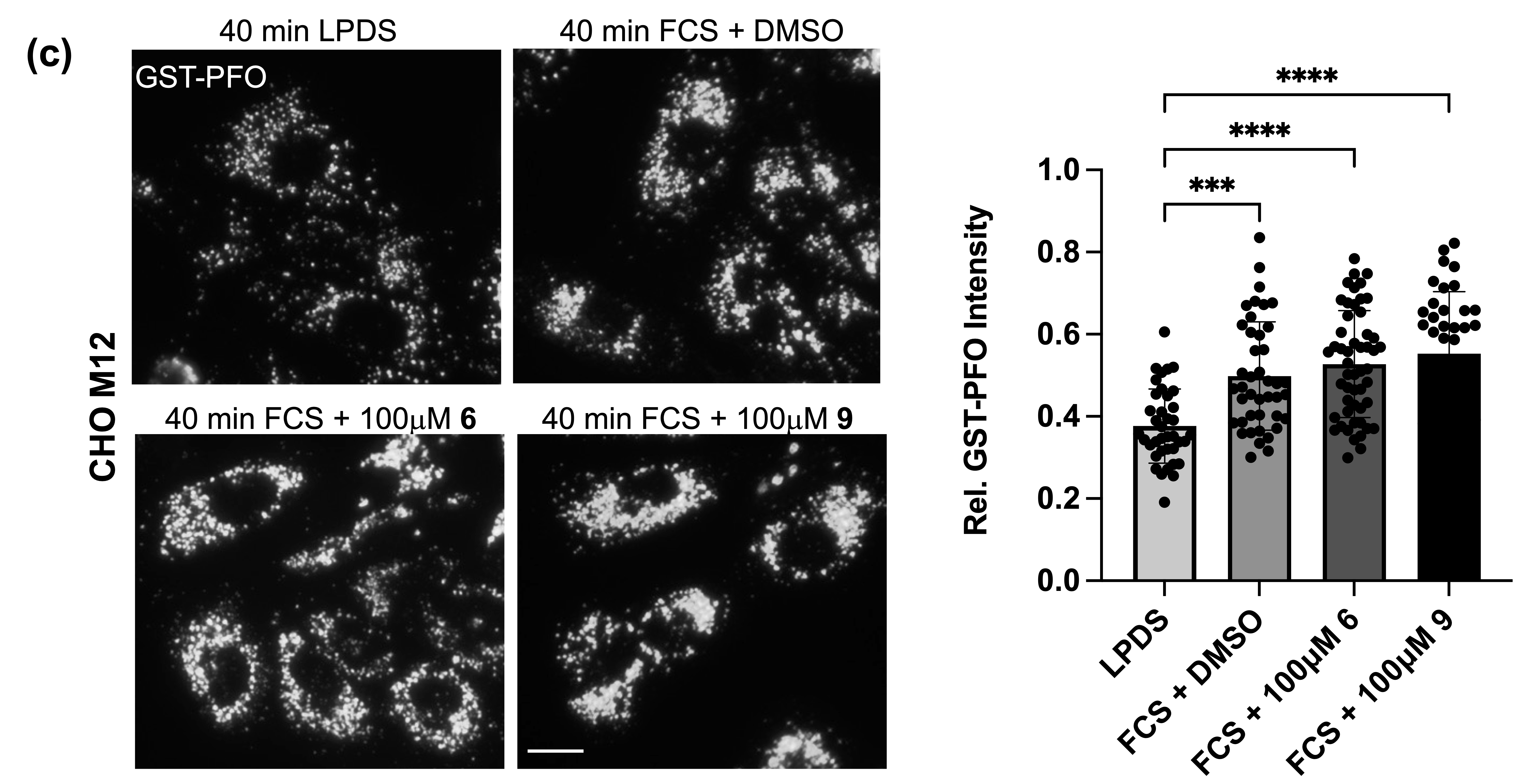
**

**Supplementary Fig. S3** Cell viability (MTS), membrane leakage (LDH) and ROS production in NPC1 mutant cells incubated with drug candidates 3, 6 and 10. 5 × 10^3^ cells were seeded in 96-well plates and grown in 10% LPDS-containing media for 48 h, followed by 24 h in media supplemented with 10% FCS ± drug candidates **3**, **6** and **10** (500 nM – 250 μM). 0.1% DMSO served as negative control. Cell viability (MTS), membrane leakage (LDH) and reactive oxygen species (ROS) production was determined (see Methods for details). **(B)** M12 cells were grown in 10% LPDS-containing media for 48 h, followed by 24 h in media supplemented with 10% FCS ± DMSO or drug candidates **3** and **9** (5 and 10 μM). Lysates were prepared and the amounts of TBC1D15, Rab7 and β-actin were determined by western blotting. Expression levels of Rab7 and TBC1D15 levels were quantified using ImageJ2 software. **(C)** M12 cells were grown in 10% LPDS-containing media for 48 h, followed by a 2 h incubation with DMSO or 100 μM compound **6** and **9**. To arrest endocytosis, cells were put on ice (4°C) for 30 min. Next, media supplemented with 10% FCS, which contains LDL-like particles, was added. To allow endocytosis, cells were incubated at 37°C for 40 min. Cells were fixed and stained for cholesterol using GST-PFO. Lipid-depleted (LPDS-treated) cells served as negative control. Representative images of cells incubated with DMSO or 100 μM compound **6** and **9** are shown. 5-8 images from each condition were captured at identical settings with fixed intensities below their saturation. Fluorescence intensity (38-52 cells/condition from 5-6 images) was determined using NIH ImageJ2 software. The mean and standard deviation (SD) is shown. GraphPad Prism 10.6 was used for statistical analysis (One-way ANOVA followed by Dunnett’s post-hoc test). *** p<0.001, **** p <0.0001. Bar is 10 μm.

**
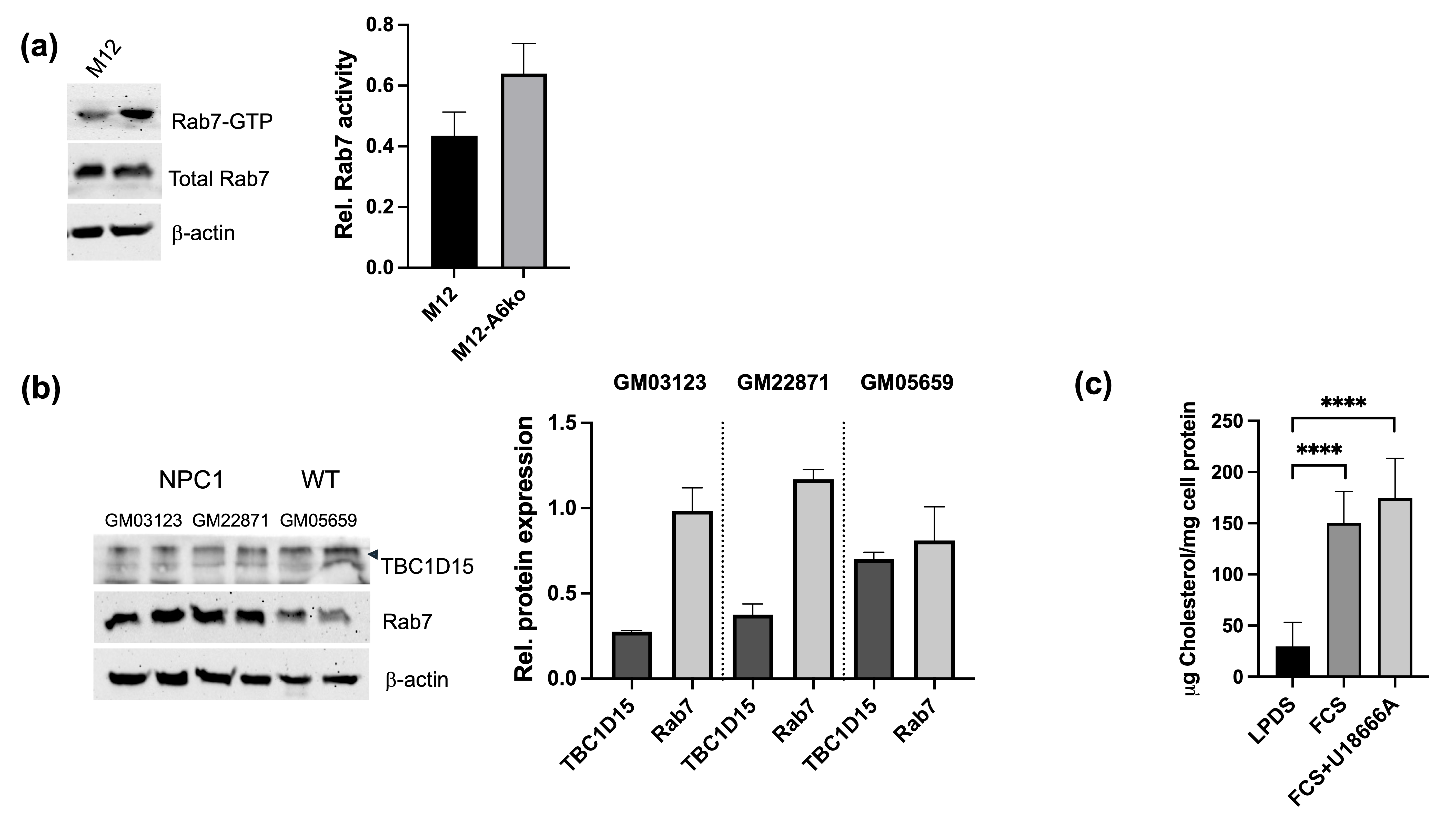
**

**Supplementary Fig. S4 (a)** Rab-GTP levels in AnxA6-depleted M12 cells. Whole cell lysates from CHO M12 and CHO M12-A6ko were prepared and Rab7-GTP pulldown assays were performed as described (see Methods for details). The amounts of active (Rab7-GTP) in pulldown samples and total Rab7 and β-actin in cell lysates were determined by western blotting and quantified (mean ± SD). Representative western blots are shown. ImageJ2 software was used for quantitative analysis of WB bands from two independent experiments. **(b)** Expression levels of Rab7 and TBC1D15 in NPC1 patient fibroblasts. Whole cell lysates from NPC1 patient (GM03123, GM22871) and control (WT) fibroblasts were prepared and the amounts of TBC1D15, Rab7 and β-actin were determined by western blotting. Expression levels of Rab7 and TBC1D15 levels were quantified using ImageJ2 software (mean ± SD). **(c)** Cellular cholesterol levels in SH-SY5Y cells. SH-SY5Y cells were grown in LPDS-containing media for 48 h. After lipid depletion, cells were grown in media with 10% FCS ± U18666A (2 μg/ml) for 24 h. Cell lysates were prepared, lipids were extracted and cellular cholesterol was measured using the Amplex Red Cholesterol Assay kit as described (see Methods for details).
